# Gastric Cancer and Microbiota: Exploring the Microbiome’s Role in Carcinogenesis and Treatment Strategies

**DOI:** 10.3390/life15070999

**Published:** 2025-06-23

**Authors:** Daniela-Cornelia Lazăr, Sorin-Dan Chiriac, George-Andrei Drăghici, Elena-Alina Moacă, Alexandra Corina Faur, Mihaela-Flavia Avram, Vladiana-Romina Turi, Mihaela-Roxana Nicolin, Adrian Goldiș, Matin Asad Salehi, Radu Jipa

**Affiliations:** 1 University Clinic of Internal Medicine IV, Faculty of Medicine, “Victor Babes” University of Medicine and Pharmacy of Timisoara, 2 Eftimie Murgu Square, 300041 Timisoara, Romania; lazar.daniela@umft.ro (D.-C.L.); chiriac.sorin@umft.ro (V.-R.T.); 2 Cardiology Department, Clinical Emergency Military Hospital, 7 Gheorghe Lazar Street, 300080 Timisoara, Romania; faur.alexandra@umft.ro; 3 University Clinic of Surgery III, Faculty of Medicine, “Victor Babes” University of Medicine and Pharmacy of Timisoara, 2 Eftimie Murgu Square, 300041 Timisoara, Romania; turi.vladiana@umft.ro; 4 University Clinic of Toxicology, Drug Industry, Management and Legislation, Faculty of Pharmacy, “Victor Babes” University of Medicine and Pharmacy of Timisoara, 2 Eftimie Murgu Square, 300041 Timisoara, Romania; 5 Research Centre for Pharmaco-Toxicological Evaluation, “Victor Babes” University of Medicine and Pharmacy of Timisoara, 2 Eftimie Murgu Square, 300041 Timisoara, Romania; 6 University Clinic of Anatomy and Embryology, Faculty of Medicine, “Victor Babes” University of Medicine and Pharmacy of Timisoara, 2 Eftimie Murgu Square, 300041 Timisoara, Romania; nicolinmihaela@yahoo.com; 7 University Clinic of Surgery I, Faculty of Medicine, “Victor Babes” University of Medicine and Pharmacy of Timisoara, 2 Eftimie Murgu Square, 300041 Timisoara, Romania; avram.mihaela@umft.ro; 8 University Clinic of Gastroenterology, Faculty of Medicine, “Victor Babes” University of Medicine and Pharmacy of Timisoara, 2 Eftimie Murgu Square, 300041 Timisoara, Romania; goldis.adrian@umft.ro; 9 Doctoral School of Dental Medicine, “Victor Babes” University of Medicine and Pharmacy of Timisoara, 9 Revolutiei 1989 Blvd., 300041 Timisoara, Romania; matin.asad-salehi@umft.ro; 10 Department of “Life Science”, Faculty of Medicine, “Vasile Goldiş” Western University of Arad, 86 Liviu Rebreanu Street, 310048 Arad, Romania; jipa.radu@uvvg.ro

**Keywords:** gastric cancer, microbiota, microbiome, *H. pylori*, carcinogenesis, treatment microbiota

## Abstract

Gastric cancer (GC) remains a major global health burden, with high morbidity and mortality rates, particularly in regions with prevalent *Helicobacter pylori* (*H. pylori*) infection. While *H. pylori* has long been recognized as a primary carcinogenic agent, recent research has underscored the broader contribution of the gastric microbiota to gastric carcinogenesis. Alterations in the microbial community, or dysbiosis, contribute to chronic inflammation, immune modulation, and epithelial transformation through a range of mechanisms, including disruption of mucosal integrity, activation of oncogenic signaling pathways (e.g., PI3K/Akt, NF-κB, STAT3), and epigenetic alterations. Furthermore, microbial metabolites, such as short-chain fatty acids, secondary bile acids, and lactate, play dual roles in either promoting or suppressing tumorigenesis. Oral and gut-derived microbes, translocated to the gastric niche, have been implicated in reshaping the gastric microenvironment and exacerbating disease progression. The composition of the microbiota also influences responses to cancer immunotherapy, suggesting that microbial profiles can serve as both prognostic biomarkers and therapeutic targets. Emerging strategies, such as probiotics, dietary interventions, and fecal microbiota transplantation (FMT), offer new avenues for restoring microbial balance and enhancing therapy response. This review synthesizes current knowledge on the complex interplay between microbiota and gastric cancer development and emphasizes the potential of microbiome modulation in both preventive and therapeutic frameworks.

## 1. Introduction

Globally, GC continues to be a major health challenge, as it is among the leading causes of cancer-related deaths, with its incidence and mortality reflecting geographical disparities, dietary habits, and underlying infections, such as *Helicobacter pylori*, considered by the World Health Organization a first-order carcinogen for gastric cancer [1,2,3,4]. According to GLOBOCAN 2022 data, GC was the fifth most common cancer worldwide, accounting for about 1 million new cases annually, with an estimated 783,000 deaths, ranking it as the third deadliest cancer after lung and colorectal cancer [5,6]. The World Health Organization (WHO) and the International Agency for Research on Cancer (IARC) indicate that the global incidence of GC is highly variable, with the highest rates typically seen in East Asia, particularly China, Japan, and South Korea. The incidence rate in these regions has been reported to be as high as 32.1 per 100,000 people, while the mortality rate can approach 13.2 per 100,000 people [6]. This pattern is also reflected in regional studies; for example, Japan has maintained one of the highest rates (27.6 per 100,000 people), attributed to dietary factors and the prevalence of *H. pylori* infections [5,6]. A high incidence is also observed in countries like Mongolia (35.5 per 100,000 people) and South Korea (27.0 per 100,000 people), underlining the influence of regional risk factors and different screening practices [7,8]. Among the leading causes of cancer-related deaths worldwide is the lack of implementation of screening programs for early detection of GC [9]. Therefore, mortality rates attributable to GC are particularly alarming in developing countries because population-based screening has not yet been widely implemented, especially in high-prevalence regions, which can contribute to the lack of early diagnosis and treatment [10]. In contrast, in developed countries, such as the United States, population-based studies have shown a downward trend in both incidence and mortality of GC in recent decades. This downward trend is due to better socio-economic conditions, decreasing rates of *H. pylori* infection, and the implementation of early detection programs [11]. In China, early detection strategies have played a significant role in improving clinical outcomes, as early detection of GC has greatly increased 5-year survival rates from 63.7 to 89%, underscoring the importance of large-scale screening programs [3]. Furthermore, downward trends in GC mortality have been observed in recent decades in regions like Taiwan and Japan, largely attributed to a combination of improved screening, improved clinical management, and a reduction in *H. pylori* infection rates [9,11,12]. Early GC often presents with nonspecific symptoms, making diagnosis possible in more advanced stages [1,13]. In addition, demographic changes, including, in particular, the aging of the population combined with the lack of progress in treatment methods, can lead to an absolute increase in mortality, despite progress in early detection practices [3,9,14]. Furthermore, this illustrates a worrying trend where younger populations (under 50) can be experiencing increasing rates of CG in contrast to the decreasing trends observed in older cohorts [9]. Although emerging research highlights that overall trends suggest a decrease in the incidence of CG, certain subpopulations, such as young cases, can show variable trends, emphasizing the importance of specific public health interventions and ongoing epidemiological surveillance [15].

Individual risk factors, such as chronic *H. pylori* infection and lifestyle choices, play a crucial role in understanding the epidemiology of GC, influencing the development and course of the disease [16,17]. *H. pylori* bacteria are implicated in chronic gastritis, peptic ulcer disease, and gastric adenocarcinoma through complex mechanisms involving chronic inflammation and direct epithelial damage [18]. The pathogenicity of *H. pylori* is mediated by its virulence factors, such as CagA and VacA, which disrupt cellular processes and promote oncogenic pathways [19]. However, emerging research emphasizes that the role of gastric microbiota in GC extends beyond *H. pylori*. The human stomach, once considered an almost sterile environment, is now known to harbor a complex and dynamic microbiota of microorganisms (gastric microbiota) that play a crucial role in health and disease. This microbiota helps digest complex dietary components and produce beneficial metabolites. It also plays an essential role in modulating immune responses and maintaining the integrity of the epithelial barrier [20,21]. The gastrointestinal microbiota, especially in the stomach, profoundly influences host immunity and the oncogenesis process. A complex microbial ecosystem interacts with the gastric mucosa to modulate local and systemic immune responses. Studies have shown that the presence of *H. pylori* and other commensal bacteria alters the composition of innate and adaptive immune cell populations, including the induction of regulatory T cells (Tregs) that are essential for the maintenance of immunological homeostasis and can exert systemic immunoregulatory effects [22,23]. When dysregulated, such immune modulation can create a micro-environment that favors chronic inflammation—a critical precursor to neoplastic transformation [24,25].

Microbial dysbiosis, a disturbance in the balance between beneficial and harmful bacteria, has been closely linked to gastrointestinal disorders, such as inflammatory bowel disease, including ulcerative colitis [20,26,27]. Recent advances in microbiome research have revealed significant changes in the composition and function of the gastric microbiota in gastric cancer patients compared to healthy individuals. In addition, the interaction between microbiota and the host immune system extends to the regulation of inflammatory cytokines and cell signaling pathways that influence epithelial barrier integrity and inflammatory responses. Dysbiosis of the gastric microbiome has been shown to promote a proinflammatory state, amplifying mucosal damage and increasing susceptibility to oncogenic transformation [28,29,30]. This state of chronic inflammation facilitates the production of growth factors and reactive oxygen species, thus contributing to genetic and epigenetic alterations in gastric epithelial cells, leading to carcinogenesis [31]. Experimental evidence supports the concept that microbiota-driven metabolic pathways and immune signaling not only modulate local inflammation but also participate in systemic cancer-promoting mechanisms [32,33].

The influence of the gut microbiota extends beyond local digestive functions. Via the gut–brain axis, microbial metabolites and signaling molecules can affect the function of the central nervous system (CNS), thereby influencing both gastrointestinal physiology and mental health [34]. In addition, there is complex bidirectional communication between gastrointestinal hormones and the gut microbiota [35]. These hormones regulate digestive processes and modulate the microbial environment, while microbial products in turn influence hormone secretion and activity. This interaction is vital for maintaining gastrointestinal homeostasis and orchestrating a robust mucosal immune response [21]. Therapeutic approaches aimed at restoring a balanced microbiota, such as the use of postbiotics and FMT, have emerged as promising interventions for restoring gut homeostasis in disease states, highlighting the critical importance of the gut microbiota not only in supporting normal gastrointestinal function but also as a potential target for alleviating a broad spectrum of gastrointestinal disorders [26,36].

In the context described above, this literature review analyzes the complex relationship between the gastric microbiota and GC, exploring the roles of specific bacteria, the impact of microbial metabolites, and potential therapeutic strategies.

In order to provide a comprehensive and up-to-date synthesis of the current understanding of the role of microbiota in gastric carcinogenesis, we conducted an extensive literature search using the electronic databases PubMed, Web of Science, Scopus, and Google Scholar. The search was focused on studies mainly published in the last 10 years, although earlier landmark studies were included where relevant for historical or foundational context. We restricted the search to articles published in English and prioritized peer-reviewed original research, systematic reviews, meta-analyses, and clinical trials, as well as articles that contained mechanistic, immunological, or therapeutic microbiota-related content. We excluded articles that reported studies unrelated to GC (e.g., other gastrointestinal tumors unless explicitly comparative), case reports without mechanistic context, non-indexed or predatory publications, as well as abstract-only or conference proceedings without full-text availability. The following keywords and combinations of terms (using Boolean operators such as AND and OR) were employed during the search: “gastric cancer”, “gastric carcinoma”, “gastric neoplasia”, “gastric microbiota”, “gastric microbiome”, “*Helicobacter pylori*”, “gut microbiota”, “oral-gastric translocation”, “dysbiosis”, “carcinogenesis”, “oncogenic pathways”, “microbial metabolites”, “short-chain fatty acids”, “bile acids”, “lactate”, “immune modulation”, “tumor microenvironment”, “immunotherapy”, “checkpoint inhibitors”, “microbiota-targeted therapy”, “fecal microbiota transplantation”, “oral microbiota”, “salivary microbiota”, “microbial biomarkers”, and “gastric dysbiosis”.

Manual screening of references from key review articles and meta-analyses was also conducted to identify additional eligible studies. The selection process focused on scientific rigor, relevance to the topic, and novelty of findings.

## 2. Helicobacter Pylori and the Gastric Microbiome

### 2.1. Role of H. Pylori in Gastric Carcinogenesis and the Microbial Dysbiosis Triggered by This Bacterium

*H. pylori* infection is a well-known risk factor for GC and also a major trigger of microbial dysbiosis in the stomach [37,38]. *H. pylori* disrupts the microbial balance in the stomach through multiple processes involving direct colonization, modulation of the host immune response, and changes in the gastric microenvironment. Following infection, *H. pylori* becomes the predominant species colonizing the gastric mucosa, effectively reducing microbial diversity, a characteristic of dysbiosis [28,39]. This reduction in diversity is attributed in part to its ability to efficiently adhere to the gastric epithelium and defeat commensal bacteria, thus altering the ecological niche that normally supports a balanced microbial community. A key mechanism underlying this disruption is *H. pylori*-induced inflammation. The bacteria trigger the secretion of proinflammatory cytokines, including IL-1β, IL-6, IL-8, and TNF-α, which contribute to mucosal damage and alter the local immune environment [40]. This inflammatory environment is driven by the host’s immune response to the bacterium’s virulence factors (CagA), creating conditions that further affect the resident commensal flora. The sustained release of cytokines and related immune mediators results in increased permeability and mucosal damage, facilitating an environment in which *H. pylori* can persist and other potentially pathogenic organisms can proliferate [40].

In addition, *H. pylori* infection induces changes in gastric physiology that contribute to microbial imbalance. The chronic inflammation associated with infection often results in reduced acid secretion due to mucosal atrophy, which in turn increases gastric pH [41]. This change in pH undermines the acid barrier that normally limits colonization by exogenous microbes, allowing overgrowth of bacteria that cannot grow under normal acidic conditions [42]. These changes in gastric acidity not only support the expansion of opportunistic pathogens but can also disrupt the natural migration and colonization patterns of bacteria that extend from the oral cavity to the stomach [43]. Furthermore, these changes can include changes in the abundance of microbial phyla, such as *Proteobacteria*, *Firmicutes,* and *Bacteroidetes*, as well as changes at the genus level, suggesting that *H. pylori* has a broad impact on community structure—an effect that has been observed when comparing *H. pylori*-positive and -negative individuals [41]. Research indicates that *H. pylori* infection results in a reduction in beneficial bacteria, such as *Lactobacillus* and *Bifidobacterium*, which are protective against inflammation and cancer. In contrast, there is an over-representation of bacteria like *Fusobacterium*, *Prevotella*, and *Streptococcus*, which are associated with proinflammatory states and can contribute to carcinogenesis [44].

**Figure 1:**
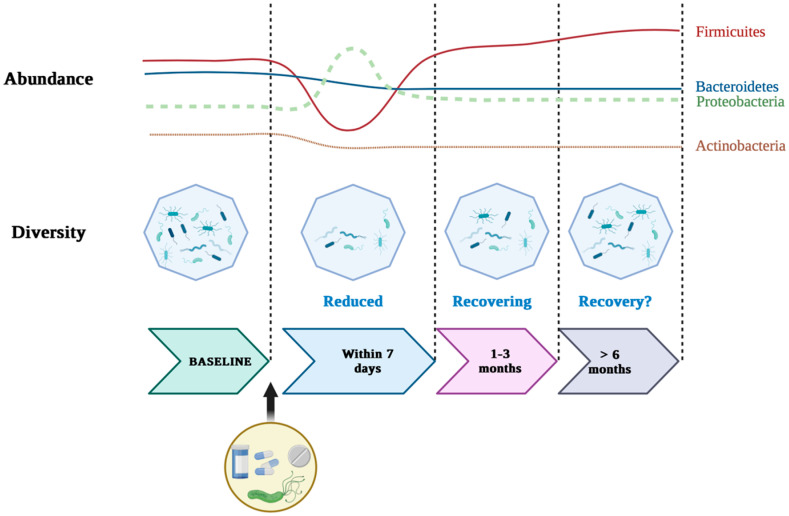
The impact of *H. pylori* eradication on the gut microbiome.

The clinical relevance of dysbiotic changes is emphasized by studies correlating microbial dysbiosis with the progression of gastric lesions. For example, the study by Liu et al. [45] was a detailed investigation of the microbial communities present in people’s stomachs with different stages of gastric disease, from chronic gastritis to advanced gastric cancer. Using next-generation sequencing, they identified key microbial agents and their interactions with *H. pylori*. Research has shown that *H. pylori* colonization significantly alters the composition and function of the gastric microbiota. In the presence of *H. pylori*, there was a notable increase in microbial diversity, including an abundance of species like *Fusobacterium* and *Prevotella*. These changes have been associated with increased inflammatory responses and cellular damage, both critical steps in gastric carcinogenesis. Microbial dysbiosis indices (MDIs) are inversely related to microbial diversity and have shown a strong association with advanced lesions, such as chronic atrophic gastritis (CAG) and intestinal metaplasia/intestinal dysplasia [22,46]. In addition, the restoration of microbial diversity after *H. pylori* eradication supports its role in stimulating dysbiosis. However, complete normalization of the gastric microbiota is not always achieved, as some reports indicate persistent dysbiotic patterns even after successful eradication therapy (Figure 1) [39,47].

Changes in the gastric microbiota following *H. pylori* eradication are partially reversible, but full recovery of microbiota composition and function appears to be incomplete and variable between individuals. Recent studies show that although eradication treatments induce temporary dysbiosis, notably by reducing the diversity and predominance of opportunistic pathogenic bacteria, some beneficial bacterial strains may recover over time [48,49]. However, the initial bacterial structure is rarely fully restored, especially in cases where patients do not follow a subsequent remodulation strategy (e.g., probiotics, diet, FMT) [50]. Thus, although diversity tends to increase a few months post-treatment, the bacterial balance is different from the initial one. Long-term studies have shown partial recovery but with a different microbiome profile from the pre-eradication status [23]. Also, the impact of antibiotic treatments on viral (virion) and fungal reservoirs is less well understood and could contribute to the instability of the microbiota in the long term [51].

**Figure 2:**
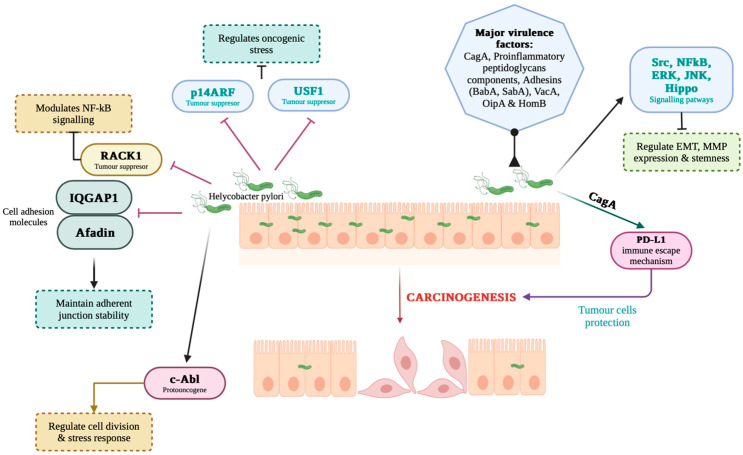
*Helicobacter pylori*-induced gastric carcinogenesis.

The role of *H. pylori* in gastric carcinogenesis is multifactorial and involves a complex interplay between chronic inflammation, genetic alterations, dysregulated signaling pathways, and epigenetic modifications. Infection with *H. pylori* induces a persistent inflammatory response in the gastric mucosa, which is a well-documented precursor to neoplastic transformation. For example, the bacteria, especially CagA-positive strains, increase the expression of proinflammatory cytokines (e.g., interleukin-8 (IL-8)) associated with increased recruitment of inflammatory cells and sustained mucosal damage [52]. This inflammatory environment not only results in chronic gastritis but also prepares the tissue micro-environment for further genetic and epigenetic changes, contributing to carcinogenesis [53]. In addition to triggering chronic inflammation, *H. pylori* manipulates various intracellular signaling cascades. The bacterium can activate mitogenic pathways, such as the gp130/STAT3 and JAK/STAT pathways in gastric epithelial cells, facilitating aberrant cell survival and proliferation [54]. In addition, *H. pylori* promotes epithelial cell survival via the PLK1/PI3K/Akt pathway and disrupts the balance between proliferation and apoptosis by inducing factors like matrilysin (MMP-7) in a virulence-factor-dependent manner [55,56]. Activation of these pathways not only prevents the apoptotic clearance of damaged cells but also encourages the accumulation of oncogenic mutations, thereby accelerating the multistep process of carcinogenesis. In addition, the bacteria’s ability to induce oxidative and nitrosative stress is essential in causing DNA damage. The thioredoxin and arginase-chaperone functions of *H. pylori* allow it to attenuate oxidative stress; however, by-products of inflammation, such as reactive oxygen species (ROS) and reactive nitrogen species (RNS), contribute to genetic alterations that lead to carcinogenesis (Figure 2) [57,58]. Disruption of tumor suppressors through phosphorylation and inactivation of phosphatase and tensin homologue (PTEN) results in further activation of the PI3K/Akt pathway, providing an additional molecular link between *H. pylori* infection and malignant transformation [59].

Another key mechanism involves the modulation of extracellular matrix remodeling and angiogenesis. *H. pylori* has been shown to increase the expression of vascular endothelial growth factor (VEGF) through several signaling pathways, including the p38 MAPK-COX-2-PGE2 MAPK-COX-2-PGE2 axis and the Wnt/β-catenin pathway [60,61]. This increase contributes to neovascularization, which is essential for tumor growth and metastasis. In parallel, the ERK/MMP9 signaling axis is involved in promoting tumor cell invasion and migration, further supporting the progression from inflammation to malignancy [62].

Epigenetically, chronic infection with *H. pylori* results in aberrant DNA methylation of CpG islands and altered microRNA (miRNA) expression patterns in gastric mucosal cells. The resulting hypermethylation silences tumor suppressor genes, while down-regulation of miRNAs, such as miR-18a-5p and miR-200a/b, contributes to down-regulation of the expression of genes essential for maintaining genomic stability [63]. These epigenetic changes underline a long-lasting memory effect of infection that predisposes even previously infected patients to gastric cancer. Animal studies further strengthen *H. pylori’s* role as an initiator of gastric carcinogenesis. Models using chemical carcinogens, such as N-Methyl-N-nitrosoureas (MNU), in the presence of *H. pylori* infection show a significant increase in the development of well-differentiated intestinal adenocarcinomas as well as preneoplastic lesions, such as intestinal metaplasia [64,65].

In this context, one can state that *H. pylori* plays an essential role in gastric carcinogenesis by establishing a chronic inflammatory state, activating oncogenic signaling pathways (e.g., STAT3, ERK, PI3K/Akt), promoting extracellular matrix remodeling and angiogenesis, and inducing epigenetic changes that inhibit tumor suppressor mechanisms. These complex processes converge to transform the normal gastric epithelium into a precancerous state and eventually into malignant lesions, thus emphasizing the importance of the bacteria as a primary risk factor for GC [52,53,54,55,56,57,58,59,60,61,62,63,64,65].

While bacterial and fungal components of the gastric microbiome have been extensively studied, the gastric virome—comprising bacteriophages, eukaryotic viruses, and endogenous viral elements—remains underexplored.

The role of the gut virome in gastric cancer is an emerging area of research, with evidence suggesting its involvement in carcinogenesis and treatment strategies. A key aspect is the potential link between the gut virome and *H. pylori* infection, a well-established risk factor for gastric cancer [66]. The role of *H. pylori* in gastric carcinogenesis is well-studied; however, the influence of the broader gut microbiome, including the virome, is an area of active investigation. Alterations in the gut virome’s composition, such as changes in the abundance and diversity of specific viral families, have been observed in inflammatory bowel diseases, and findings related to colorectal cancer suggest potential parallels in other gastrointestinal disorders [67,68].

Studies have reported that the gut virome of patients with gastrointestinal diseases, including GC, can significantly differ from that of healthy individuals [68,69,70]. For example, a study indicated a shift from virulent to temperate phages in colorectal cancer patients, suggesting a role for specific phages in carcinogenesis [68]. Furthermore, the gut virome interacts with the gut bacteriome, and these interactions may have implications for GC development and progression. Bacteriophages, which make up a significant portion of the gut virome, are known to regulate bacterial diversity and metabolism [70]. Changes in the virome, including shifts in bacteriophage populations, may lead to dysbiosis in the gut’s bacterial community, potentially contributing to gastric cancer [66]. Additionally, the gut virome may influence the host’s immune response and inflammation, critical factors in gastric cancer pathogenesis. Research indicates that the gut virome can modulate the host immune system, which may lead to chronic inflammation and increased susceptibility to cancer [71].

Despite numerous studies evaluating microbiota changes after *H. pylori* eradication, results remain inconsistent [72]. Some report partial restoration of microbial diversity [47,49,73], while others show persistent dysbiosis [48,74,75]. These discrepancies likely stem from variations in follow-up duration, sample types (gastric vs. fecal), and geographic differences in microbiota composition. Additionally, antibiotic regimens and patient-specific factors, such as diet and age, are often not uniformly controlled, limiting comparability across studies. Yet, data regarding the long-term reversibility of dysbiosis after *H. pylori* eradication remain inconsistent. While some studies demonstrate partial restoration of microbial diversity post-treatment, others report persistent dysbiotic patterns and functional instability, suggesting that early eradication may be crucial in preventing irreversible ecological shifts.

### 2.2. Synergistic and Antagonistic Interactions with Other Microbes

As stated above, the mechanisms through which *H. pylori* influences the gastric environment are pH alteration and secretion of virulence factors, which in turn affect other microbial species. The synergistic interactions between *H. pylori* and these microbes exacerbate mucosal inflammation and promote genetic instability in gastric epithelial cells. This complex microbial ecosystem creates a favorable microenvironment for cancer growth. *H. pylori* establishes itself as the dominant species upon colonization and modulates the overall microbial network, leading to both cooperative and competitive interactions between different microbial taxa. One aspect of these interactions is antagonism. Under healthy gastric conditions, several commensal taxa contribute to maintaining mucosal integrity and immune homeostasis. However, *H. pylori* colonization can lead to antagonistic interactions with certain bacterial genes. For example, Guo et al. [22] identified that *Neisseria* and *Prevotella*, which are usually found in a healthy gastric microbiota, exhibit antagonistic patterns with *H. pylori* in advanced lesions. Such interactions suggest that the depletion of these bacteria can remove a protective influence within the gastric ecosystem, thereby facilitating a microenvironment favorable to chronic inflammation and neoplastic progression. Suppression of potentially protective commensals by *H. pylori* can be mediated by competitive nutrient uptake, altered pH conditions, or immune modulation, ultimately compromising the ability of the host to limit procarcinogenic changes [22]. Therefore, the disruption of microbial homeostasis through *H. pylori* colonization can create an environment conducive to carcinogenesis. Reduced protective bacteria and increased pathogenic species can exacerbate mucosal inflammation and contribute to the progression of gastric lesions. This microbial dysbiosis highlights the potential of microbiota targeting as a therapeutic strategy in preventing GC.

Conversely, synergistic interactions are also evident. Studies by Coker et al. [76] have shown that the dysbiosis observed during gastric tumorigenesis involves the enrichment of oral bacterial taxa that form specific niche interactions with *H. pylori*. These synergistic relationships can enhance inflammatory responses; for example, the interaction between *H. pylori* and certain oral microbes can contribute to increased production of inflammatory mediators, further promoting a carcinogenic environment. Complementary studies have also observed alterations in extragastric microbial communities, such as those found on the tongue coating, suggesting that *H. pylori*-induced dysbiosis can have a systemic component [77]. Liu et al. [45] reported how *H. pylori* can interact with other microbial species to potentiate carcinogenesis. In this context, *H. pylori* can collaborate with other bacteria through metabolic cooperation that exacerbates mucosal lesions and sustains chronic inflammation. Das et al. [41] have further demonstrated that microbial interaction networks in *H. pylori*-infected individuals often include bacteria that can synergize with *H. pylori*, potentially by amplifying inflammatory signals or disrupting epithelial barriers, thereby accelerating mucosal transformation towards malignancy.

The dual nature of these microbial interactions—antagonistic exclusion of beneficial taxa and synergistic collaboration with opportunistic pathogens—illustrates the complexity of the gastric ecosystem. Furthermore, these interactions are dynamic and context-dependent. In the initial stages of colonization, some commensals can resist *H. pylori* establishment; however, as chronic infection persists, *H. pylori*-driven dysbiosis becomes more pronounced, leading to a restructured microbial community that favors carcinogenic processes [22,76]. This restructuring not only affects local immune responses but also alters the metabolic output of the gastric microbiome, creating conditions that favor neoplastic transformation.

*H. pylori* does not act in isolation but interacts dynamically with other microbial species in the gastric environment. Some commensals exhibit synergistic relationships that may exacerbate pathogenic effects, while others antagonize *H. pylori*, potentially mitigating its impact. These interactions can influence colonization patterns, immune modulation, and progression towards gastric pathology, underscoring the complexity of microbial networks in gastric carcinogenesis. Nonetheless, caution is needed in interpreting these associations. Current evidence is largely correlative, with few mechanistic studies confirming causation. For instance, the co-occurrence of oral-type microbes in GC patients may reflect ecological displacement rather than a causal role. Future studies should use longitudinal sampling and functional assays to address this limitation.

## 3. Gastric Microbiota Alterations in Carcinogenesis

### 3.1. Microbiota Composition in Precancerous and Cancerous Lesions

The complex relationship between gastric microbiota and GC has attracted significant attention, particularly in the context of its alterations during precancerous stages. Recent research reported by Zhang et al. [38] highlights dynamic changes in microbial communities that precede and accompany gastric carcinogenesis. A key observation is the dominance of *H. pylori* in people with chronic gastritis and atrophic gastritis, conditions recognized as precursors of GC. Precancerous gastric lesions, such as atrophic gastritis and intestinal metaplasia, are critical steps in the process of gastric carcinogenesis. These lesions are accompanied by distinct changes in the composition of the gastric microbiota, which serve both as biomarkers and potential mediators of disease progression. Infection with *H. pylori* is intrinsically linked to the development of precancerous lesions, as its virulence factors (CagA, VacA) significantly promote chronic inflammation that predisposes the gastric mucosa to neoplastic transformation [78]. In the early stages of *H. pylori*-associated gastritis, the bacteria dominate the gastric ecosystem, resulting in reduced overall microbial diversity. However, when progression to atrophic gastritis and intestinal metaplasia occurs, the microenvironment undergoes notable physicochemical changes, including an increase in gastric pH due to loss of function of the parietal acid-secreting cells. Such modifications provide a permissive environment for colonization by a wider range of microbial taxa [47,79].

Several studies have elucidated that with the development of atrophy and metaplasia, the composition of the gastric microbiota shifts from a community dominated by *H. pylori* to one enriched with gut-type bacteria. Meta-analysis data indicate that key bacteria, such as *Firmicutes*, *Bacteroidetes*, *Actinobacteria,* and *Proteobacteria,* are becoming more prominent, reflecting changes in available nutrients and altered mucosal defense mechanisms [80]. These findings suggest that as the severity of mucosal lesions increases, the previously predominant *H. pylori* population decreases, allowing opportunistic and commensal bacteria in the oral cavity and the intestines to establish themselves [81]. In addition, studies comparing the microbiota among patients with different stages of precancerous lesions have reported that in contrast to non-lesional tissues, areas with intestinal metaplasia show an over-representation of bacteria typically associated with the intestinal environment [46]. This change not only evidences a remodeling of the gastric microbiome but also correlates with increased levels of proinflammatory mediators and potential genotoxic metabolites, which can further promote progression from inflammation to neoplasia. Furthermore, systematic reviews have reported that certain taxa, such as *Veillonella* and *Leptotrichia*, are enriched in the gastric microbiota of gastric cancer patients, whereas other taxa, such as *Neisseria*, are reduced relative to non-cancer controls [28]. These findings suggest that an imbalance (dysbiosis) plays a role in the microenvironmental changes that predispose the gastric mucosa to neoplastic transformation.

**Figure 3:**
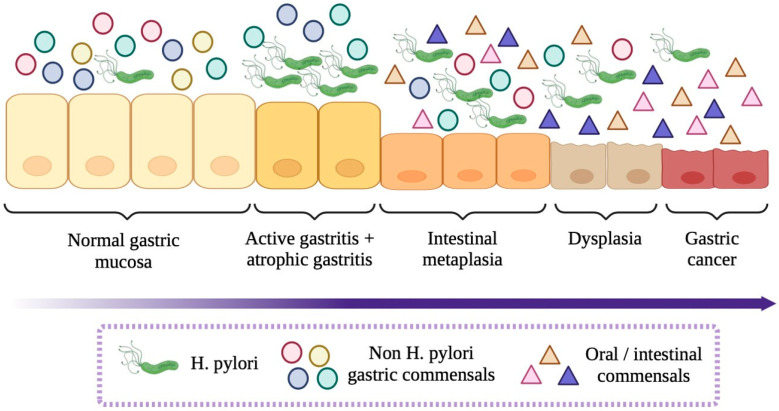
*H. pylori* and non-*H. pylori* microbes in the development of gastric carcinogenesis.

In addition to these taxonomic changes, research using molecular techniques, such as 16S rRNA gene sequencing, has shown that patients with gastric cancer tend to have a smaller microbial community and altered interaction networks compared to people with non-cancerous gastric conditions [82]. For example, *Lactobacillus* dominance was observed in gastric cancer samples, indicating that community restructuring involves not only loss of diversity but also opportunistic expansion of bacteria that can have pro-carcinogenic properties [82]. In addition, comparisons of mucosa-associated microbiota between cancerous and adjacent non-cancerous tissues emphasized the presence of niche-specific microbial networks that are uniquely disrupted in gastric cancer patients [83]. These findings suggest that interbacterial interactions, as well as the functional capacity of the altered microbiome, are substantially related to the tumor microenvironment, potentially influencing tumor progression by modulating local immune responses and metabolic processes. Meta-analytic approaches further support the observation that microbial dysbiosis, including both a reduction in overall diversity and specific compositional changes, is a hallmark of gastric carcinogenesis [80]. Collectively, these studies reinforce the idea that changes in microbial diversity in GC patients are not just by-products of tumorigenesis but can actively contribute to the pathophysiological processes underlying cancer development (Figure 3).

### 3.2. Fungal Microbiota and Bacterial–Fungal Interactions

The fungal components of the gastric microbiota, although less abundant than bacterial homogenates, are increasingly recognized as important factors in the complex ecosystem of the human stomach. In addition to the dominant bacteria, fungal species, such as *Candida albicans,* are frequently present. This observation challenges the previously held idea that *H. pylori* is uniformly dominant in gastric niches and emphasizes the relevance of fungi in modulating the microbial ecology. The gastric microbiome, including, in particular, colonization by *Candida albicans* and related fungal species, can contribute to the tumor microenvironment and influence the pathogenesis of gastric cancer. Fungi are important factors in modulating local inflammation, epithelial integrity, and carcinogenic processes. Chronic colonization by *C. albicans* can exacerbate mucosal inflammation and promote a state of persistent epithelial lesion that predisposes to malignant transformation. In animal models, *C. albicans* has been shown to invade not only the esophagus but also gastric tissues, evoking hyperkeratosis and a robust inflammatory response dominated by granulocytes [84]. The invasive properties and tissue tropism of *C. albicans*, in contrast to strains like *Candida glabrata*, highlight the potential of certain fungal species to disrupt the mucosal barrier and trigger proinflammatory signaling cascades that can synergize with other risk factors for carcinogenesis.

**Figure 4:**
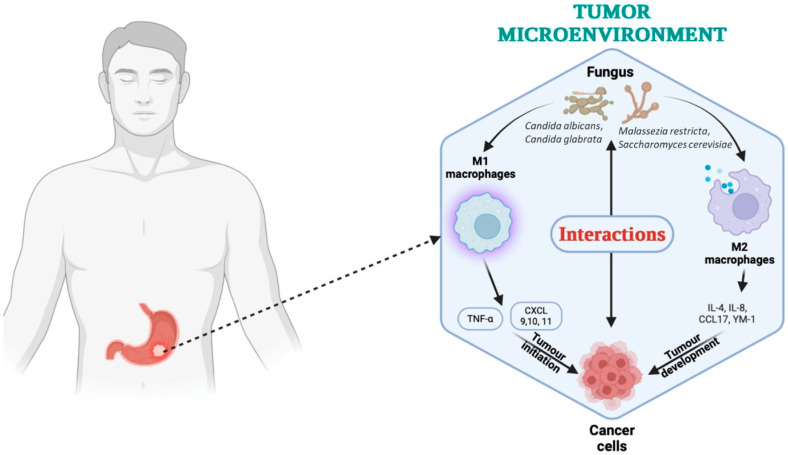
The interactions between specific fungi and immune factors in the tumor microenvironment.

Clinical observations further support the role of gastric fungi in the context of tumor progression and postoperative complications. For example, the case report by Kertmen et al. [85] described a case of purulent pericarditis with cardiac tamponade secondary to *Candida albicans* dissemination following gastric carcinoma surgery. Such findings imply that not only fungal overgrowth can contribute to gastric mucosal lesions, as systemic dissemination of fungal elements can also complicate the clinical course of GC patients, suggesting an interplay between local fungal dynamics and overall disease prognosis. Studies have also shown that alterations in gastric pH because of Crohn’s gastritis, atrophic changes, or surgery, such as vagotomy, can predispose to *Candida* overgrowth. Brooks et al. [86] demonstrated that an increase in gastric juice pH after vagotomy is associated with a high incidence of intraluminal growth of *C. albicans*. This observation reinforces the idea that under normal acidic conditions, the gastric environment limits fungal colonization; however, when acid secretion is impaired, the resulting fungal overgrowth can create a microenvironment favorable to chronic inflammation and tumorigenesis (Figure 4).

In addition to *C. albicans*, other fungal species have been increasingly implicated in modulating immune responses within the gastric tumor microenvironment. *Malassezia restricta*, a lipophilic yeast commonly found on skin and mucosal surfaces, has been detected in gastrointestinal tumors and is associated with activation of the complement cascade and C3 signaling, promoting inflammation and immune evasion. Furthermore, *Candida glabrata*, often co-colonizing the gastric mucosa under dysbiotic conditions, may contribute to epithelial barrier dysfunction and immune modulation, although its pathogenicity is less direct compared to *C. albicans*. Another emerging genus is *Saccharomyces*, particularly *S. cerevisiae*, which may participate in dysregulated immune crosstalk when present in altered fungal communities [87].

These species, through direct interactions with host immune cells or by shaping the bacterial microbiota, are integral to the dynamic crosstalk occurring in the tumor microenvironment and deserve further investigation as potential immunomodulatory agents or biomarkers in gastric cancer [88].

Although *C. albicans* has been linked to gastric mucosal damage and biofilm synergy with bacteria, the evidence is largely correlative. Many studies rely on fungal detection via non-targeted methods [89,90,91], and few have explored mechanistic pathways [92]. Moreover, it remains unclear whether fungal overgrowth is a cause or consequence of gastric dysbiosis. Experimental heterogeneity and limited sample sizes further constrain definitive conclusions. Although less studied than bacteria, fungal species, particularly *C. albicans*, may contribute to gastric carcinogenesis through synergistic biofilm formation, mucosal barrier disruption, and proinflammatory effects. Their interactions with bacterial microbiota can amplify immune dysregulation. However, current data remain limited and largely associative, necessitating more mechanistic studies on the gastric mycobiome.

## 4. Microbial Metabolites and Oncogenic Pathways

**Figure 5:**
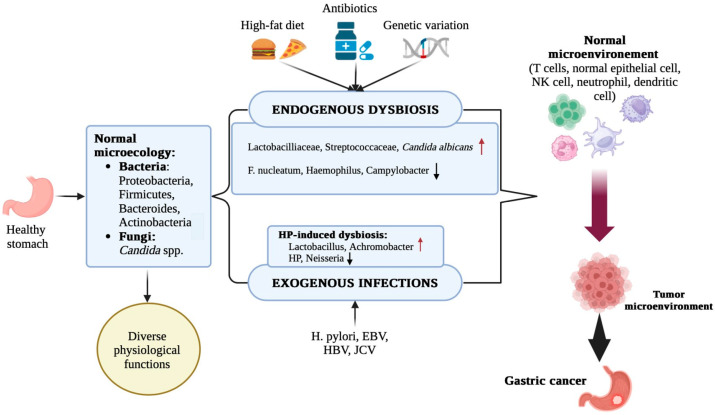
Factors affecting gastric microecologic dysbiosis and potential mechanisms underlying microecologic dysbiosis causing gastric carcinogenesis.

Stomach mucosa-associated microbiota undergo significant changes in patients with gastric cancer, contributing to the pathogenesis of the disease [83,93]. This microbial community, which interacts closely with the gastric mucosa, plays a crucial role in maintaining gastric health and influencing disease states [94]. Microbial changes are often accompanied by changes in gastric metabolites. Alterations in the gastric microbiota lead to changes in the production of key metabolites, including short-chain fatty acids (SCFAs), lactate, bile acids, polyamines, and N-nitroso compounds, which can modulate inflammatory pathways, epigenetic regulation, and cellular metabolism in the tumor microenvironment [66]. Figure 5 shows the factors that affect gastric microecologic dysbiosis and the potential mechanisms underlying the microecologic dysbiosis causing gastric carcinogenesis.

The diversity and composition of the gastric microbiota differ significantly between healthy people and those with GC. In GC patients, there is a notable decrease in microbial diversity and a change in microbial community structure. Specifically, there is a reduction in genera of beneficial bacteria, such as *Lactobacillus*, and an increase in potentially pathogenic bacteria, such as *Fusobacterium* and *Peptostreptococcus*. *H. pylori* remains a key factor, but altered microbiota also contribute to carcinogenesis independently of *H. pylori* [93,94]. These changes in the microbiota can lead to a proinflammatory state, increased production of harmful metabolites, and promotion of genetic mutations. Altered microbiota can influence the immune response, epithelial integrity, and local inflammation, creating a microenvironment favorable for cancer development.

### 4.1. Key Microbial Metabolites

The interaction between gut microbiota and GC has attracted increasing attention, particularly in relation to how microbial metabolites, such as butyrate, acetate, and secondary bile acids, modulate key carcinogenic pathways. These metabolites are not only by-products of microbial metabolism but also dynamic modulators of host cell functions that affect tumor initiation and progression.

SCFAs, such as acetate, propionate, and butyrate, are generated by the fermentation of dietary fiber by certain gut bacteria, and they are well-known for their immunomodulatory and anti-inflammatory properties. One of the main metabolites, butyrate, functions as a histone deacetylase (HDAC) inhibitor and thus induces epigenetic changes that can reactivate tumor suppressor genes while reducing oncogene expression [95,96]. Acetylated histone accumulation alters transcriptional profiles in gastric epithelial cells and contributes to modulating immune responses, facilitating anti-inflammatory effects, and promoting apoptosis of transformed cells [96]. As for butyrate, emerging evidence supports its potential in the treatment of gastric cancer, where epigenetic regulation is essential for tumoral behavior [95,97]. This highlights the dual role of butyrate both in cancer prevention and as a supportive treatment by remodeling the tumor microenvironment.

Acetate, another SCFA generated through microbial fermentation, has been implicated in driving metabolic and immune pathways in the tumor environment. Investigations using sensitive analytical techniques, such as ion-selective flow tube mass spectrometry, have shown that vapors from the gastric cavity of cancer patients have higher average levels of acetic acid compared to non-cancer cohorts [98]. This metabolite plays an important role in cellular energy metabolism, serving as a substrate for the production of acetyl-CoA, which is essential for lipid biosynthesis and the metabolic requirements of proliferating tumor cells [98]. In addition, acetate has been associated with the restoration of T cell immunity, an effect that has been shown in studies of other tumor types, suggesting its importance in counteracting tumor-induced immunosuppression [99].

Lactate, long considered only a metabolic by-product of anaerobic glycolysis, has emerged as a pivotal signaling molecule orchestrating both immune evasion and tumor proliferation. In cancer cells, the Warburg effect leads to high lactate production even under aerobic conditions, leading to its accumulation in the tumor microenvironment (TME) [100]. This accumulation contributes to an acidic environment that impairs the function of immune effector cells, such as CD8+ T cells and natural killer (NK) cells, thus facilitating immune evasion [101,102]. At the molecular level, lactate modulates the TME through multiple mechanisms. For example, lactate can induce histone lactylation—a novel post-translational modification that directly influences gene expression in both tumor cells and tumor-associated immune cells [103]. This lactylation not only promotes metabolic reprogramming that supports tumor cell proliferation but also shifts tumor-associated macrophages to an immunosuppressive M2 phenotype [104]. Such reprogramming facilitates the release of cytokines and growth factors that further enhance tumor growth and metastasis [104]. In addition, lactate signaling through receptor-mediated pathways strengthens immunosuppressive networks by increasing the activity of regulatory T cells while simultaneously preventing cytotoxic responses, thereby enhancing immune evasion [102,105]. Beyond immune modulation, lactate serves as an essential metabolic substrate that fuels tumor proliferation. It is transferred between cells within the TME, acting as an alternative energy source and supporting anabolic processes essential for rapid cell division [100]. This dual functionality, providing direct metabolic support to tumor cells while altering the local immune landscape, creates a vicious cycle in which lactate promotes both cell proliferation and protects tumor cells from immune-mediated killing [105]. The interplay of these processes underlines the importance of lactate in stimulating tumor progression and highlights its potential as a therapeutic target, where strategies to disrupt lactate production or signaling could restore immune function and inhibit tumor growth [102,105].

Recent research has highlighted the complex relationship between bile acids, the microbiome, and gastric carcinogenesis. Wang et al. [106] have demonstrated how interactions between bile acids and the microbiome can significantly influence the development of GC. Bile acids, produced in the liver and secreted in the intestine, play a crucial role in the digestion and absorption of fats. However, their role extends beyond digestion; they also act as signaling molecules that influence various metabolic pathways. In the stomach, bile acids can modulate the composition and function of the gastric microbiome. This interaction can lead to significant changes in the microbial community, contributing to a pro-carcinogenic environment. The conversion of primary bile acids to secondary bile acids by the gut and the gastric microbiota is a critical step linking bile acid metabolism to DNA damage and eventual malignant transformation of gastric epithelial cells [106,107]. Under pathologic conditions (such as bile reflux, postoperative changes, or dysbiosis of the microbiota), bile acids can accumulate in the gastric environment, thereby increasing the exposure of the gastric mucosa to these cytotoxic and genotoxic agents [106,107].

Secondary bile acids, produced through microbial conversion of primary bile acids, are another important class of metabolites with a potential procarcinogenic role in GC. Although the literature on secondary bile acids in the gastric context is still emerging, data indicate that these molecules may interact with nuclear receptors and other signaling molecules to promote proinflammatory responses, DNA damage, and subsequent cellular transformation [108]. Such interactions may activate or suppress downstream pathways governing cell proliferation and apoptosis, integrating microbial dysbiosis with the molecular pathogenesis of gastric carcinogenesis [108]. Once formed, secondary bile acids, such as deoxycholic acid (DCA) and chenodeoxycholic acid, exhibit strong hydrophobic properties that facilitate cellular uptake and intracellular accumulation [106,109]. This accumulation is associated with increased generation of ROS that causes oxidative stress and promotes DNA damage. In different cellular models, it has been shown that DCA induces oxidative DNA damage, such as 8-oxoguanine adducts, which compromises genomic integrity and may lead to carcinogenesis [107,110]. The resulting DNA chain breaks, mutations, and subsequent genomic instability create a cellular environment favorable for neoplastic transformation [110,111]. Bacterial modification of bile acids not only leads to the formation of these strong secondary bile acids but also creates a proinflammatory environment [106]. This inflammatory microenvironment, enriched in cytokines and stress-responsive signaling molecules, further exacerbates oxidative DNA damage and tissue damage, thereby lowering the threshold for malignant conversion [106,107,111]. In addition, chronic exposure of gastric epithelial cells to these compounds stimulates compensatory proliferative and anti-apoptotic pathways, contributing to the persistence and propagation of genetically compromised cells and thus promoting the progression of premalignant lesions to GC manifestation [106,109]. The deleterious effects of these secondary metabolites underscore complex host–microbe interactions in the progression of gastric malignancies. In parallel, microbial dysbiosis may influence bile acid metabolism. Intestinal bacteria modify primary bile acids into secondary bile acid species, including DCA and lithocholic acid, which are involved in lipid absorption and have been implicated in carcinogenic processes through their effects on cell signaling and inflammation pathways [112]. These bile acid metabolites can also interfere with host lipid homeostasis, further linking metabolic dysregulation to gastric tumorigenesis.

This wide range of metabolites, which influence GC, has both protective and harmful roles. Certain metabolites endogenously produced or derived from food sources exhibit anti-inflammatory and chemopreventive properties, while others contribute to carcinogenic processes by promoting chronic inflammation and cell transformation.

Anti-inflammatory metabolites can act as effective chemopreventive agents in GC. For example, α-difluoromethylornithine (DFMO) has been shown to reduce inflammation-associated gastric tumorigenesis by modulating ornithine decarboxylase activity, thereby reducing excessive polyamine synthesis linked to cell proliferation and inflammation [113]. In addition, dietary phytoactives, such as curcumin and organosulfides found in cruciferous vegetables, have demonstrated the ability to induce cell cycle arrest, promote apoptosis, and inhibit inflammatory mediators that are pivotal in gastric tumor progression [114,115,116]. Complementarily, butyrate-producing bacteria in the gut generate short-chain fatty acids that support intestinal homeostasis by secreting anti-inflammatory molecules and promoting an environment that mitigates carcinogenic risks [117]. These findings underline the potential to target beneficial metabolites or their producers as a preventive strategy against inflammation-induced gastric carcinogenesis.

Certain metabolites exhibit pro-carcinogenic properties by exacerbating inflammatory responses and promoting tumor growth. Secondary bile acids, for example, have been implicated in gastric carcinogenesis through their interaction with receptor-mediated pathways that enhance proinflammatory signaling and DNA damage [118]. Chronic exposure to such metabolites can disrupt the integrity of the mucosa and create an environment favorable for malignant transformation. In addition, microbiota–host interactions leading to overproduction of cancer-regenerative compounds, combined with a deregulated inflammatory response, further support the role of these metabolites in GC progression [119]. The simultaneous existence of these opposing roles emphasizes that the balance between protective and harmful metabolites is essential. Perturbation of this balance, influenced by factors like diet, microbial composition, and genetic predisposition, creates a scenario in which the trophic influence of anti-inflammatory and anticarcinogenic compounds is overwhelmed by persistent, low-grade inflammation triggered by pro-carcinogenic metabolites [120].

Therefore, the dual role of metabolites in GC exemplifies the complexity of host–microbe interactions and the multifactorial nature of tumorigenesis. Therapeutic strategies can benefit from a holistic approach that not only suppresses the production or action of carcinogenic metabolites but also promotes the biosynthesis of anti-inflammatory agents. Further investigations of this metabolite balance are needed to refine these strategies and develop personalized interventions that could restore a protective metabolic environment in the gastric mucosa.

### 4.2. Metabolite-Induced Signaling in Cancer Cell Proliferation and Inflammation–the Role of Specific Metabolites as Diagnostic and Prognostic Markers

Metabolite-induced signaling plays an essential role in mediating GC cell proliferation and inflammation through concerted actions of microbial and host-derived metabolites. Emerging evidence suggests that deleterious bacterial metabolites generated in a dysbiotic gastric microenvironment serve as potent activators of oncogene signaling pathways. For example, chronic exposure to ROS, which are by-products of chronic inflammation, has been linked to increased cell proliferation through the activation of mitogenic cascades, such as the MAPK pathway, thereby promoting neoplastic transformation [121]. These ROS and other bacterial-derived metabolites are thought to create an augmentation loop whereby sustained cellular stress results in further deregulation of cell growth controls. A key pathway mediating the effects of these metabolites is the cyclooxygenase (COX) pathway. In COX-2 expressing GC cells, ω-6 polyunsaturated fatty acids (PUFAs) and their metabolite prostaglandin E2 (PGE2) have been shown to significantly increase cell proliferation, invasion, and angiogenesis [122]. This mechanism is driven by the binding of PGE2 to its receptors on gastric epithelial cells, which in turn activate downstream effectors, such as the cAMP/PKA and MAPK signaling cascades. These pathways converge to promote the proliferation of malignant cells as well as remodeling of the tumor microenvironment in a way that favors neoplastic progression. In parallel, bacterial lipopolysaccharide (LPS) and other pathogen-associated molecular patterns (PAMPs) can engage the NF-κB signaling pathway in gastric epithelial cells. Upon recognition by Toll-like receptors (TLRs), these microbial molecules induce a robust inflammatory response characterized by the production of proinflammatory cytokines, which further stimulate cancer cell proliferation and survival [123]. Activation of NF-κB, involved in gastrointestinal tumorigenesis, acts as a transcriptional hub for inflammatory mediators that enhance the local proinflammatory environment and facilitate the avoidance of apoptosis by cancer cells. In addition, secondary metabolites produced by the gut microbiota, including SCFAs, indoles, and bile acid derivatives, contribute substantially to the regulatory landscape by modulating epigenetic changes and influencing signal transduction pathways. These metabolites can act as signaling ligands, binding to certain G-protein-coupled receptors (GPCRs) or nuclear receptors, such as the aryl hydrocarbon receptor, and thereby altering gene expression patterns that govern cell cycle progression and immune responses [96]. Butyrate, for example, can induce apoptosis in aberrant cells; however, in the context of a perturbed microenvironment, its concentration and local activity can be insufficient to prevent malignant transformation.

Metabolomic profiling has emerged as a powerful tool to elucidate the complex metabolic alterations occurring in patients with GC. These studies use advanced technologies, such as gas chromatography/mass spectrometry (GC/MS), liquid chromatography/mass spectrometry (LC-MS), nuclear magnetic resonance spectroscopy (NMR), and mass spectrometry imaging, to capture global and spatial metabolic changes in gastric tissues and serum. Metabolomic analyses performed in GC patients identified a diverse range of metabolites discriminating between tumor and non-tumoral tissues, suggesting that localized changes in microbial activity can lead to distinct metabolic signatures depending on tumor location (proximal versus distal) [124]. This evidence indicates that microbial metabolites not only serve as biomarkers for disease state but can also play active roles in GC progression through mechanisms like modulation of the immune response, alteration of intestinal barrier function, and direct effects on epithelial cell proliferation.

Song et al. [125] have used GC/MS to discover that gastric cancer cells use a large amount of cholesterol for biomembrane synthesis and exhibit increased glycolysis with disruption of the tricarboxylic acid (TCA) cycle. Their data demonstrated distinct changes in metabolite intermediates, such as fumaric acid and α-ketoglutaric acid, suggesting a robust reprogramming of energy metabolism. Complementarily, direct metabolomic studies by Kaźmierczak-Siedlecka et al. [126] identified specific plasma metabolite signatures—including changes in lipids and fatty acids—that differentiate patients with GC from individuals with benign gastric lesions and indicate potential biomarkers for early detection of the disease. Furthermore, by using high-throughput mass spectrometry and NMR-based techniques, researchers have delineated distinct metabolic signatures associated with GC compared to non-cancerous controls [127]. These profiling studies facilitate the identification of conserved and aberrant metabolic pathways, thus highlighting potential biomarkers for early detection and therapeutic targets.

In addition to serum profiling, tissue metabolomics provided a more localized picture of metabolic dysregulation within the tumor microenvironment. Tissue fingerprinting studies by Song et al. [128] using GC/MS indicated severe disruptions in energy metabolism and lipid biosynthesis in cancerous tissues compared to adjacent normal mucosa. In addition, imaging mass spectrometry approaches have revealed metabolic spatial heterogeneity within gastric tumors, confirming the presence of different metabolic phenotypes that may reflect variable microenvironments [129].

Developing the analysis of individual metabolites, several studies have explored specific metabolic pathways and their roles in GC progression. Hong et al. [130] reported that abnormal arginine synthesis is associated with worse prognosis in patients with stage three GC, highlighting the potential of amino acid metabolism as a prognostic biomarker. Meanwhile, investigations into tryptophan metabolism have demonstrated that tryptophan and its derivatives in serum and gastric juice can serve as noninvasive biomarkers for early detection of GC [131]. In a parallel study, Deng et al. [132] identified elevated levels of aromatic amino acids in gastric juice during early stages of cancer progression, further supporting the utility of metabolomic profiling for early diagnosis.

Multiomics approaches have integrated metabolomic profiling with machine learning techniques to refine diagnostic models for GC, thereby advancing precision medicine strategies. Such approaches combine data from genomic, proteomic, and metabolomic analyses to capture the complex metabolic reprogramming inherent in GCs [133]. This integration not only enhances the sensitivity and specificity of diagnostic biomarkers but also provides mechanistic insights into the interplay between metabolomics and oncogenic processes. In this context, metabolomic studies using gas chromatography/mass spectrometry have differentiated between malignant and non-malignant gastric tissues, highlighting the clinical utility of these metabolic biomarkers in the early detection of GC [127]. Plasma metabolomics and lipidomic studies also contributed valuable information. For example, Wang et al. [134] demonstrated that early GC is accompanied by significant reductions in specific lipid metabolites, such as triacylglycerols, indicating disturbances in lipid metabolism. Yu et al. [135] and Bu et al. [136] further extended these findings by applying non-targeted metabolomic techniques and analyzing extracellular vesicle-derived metabolites, respectively. These studies have not only improved the sensitivity and specificity of early detection of GC but have also helped to elucidate the interplay between systemic metabolic changes and local tumor biology.

Integrative studies have combined metabolomic data with other omics layers, such as microbiomics and transcriptomics, to elucidate multifaceted interactions between the gastric microbiota and host metabolism in GC [37,137]. For example, targeted metabolomics coupled with 16S rRNA sequencing revealed unique microbiome–metabolite interactions that correlate with progression from superficial gastritis to intestinal metaplasia and, ultimately, to GC [137]. The integration of metabolomic data with other omics layers has allowed for a more in-depth understanding of the interplay between genetic alterations, metabolic reprogramming, and the tumor microenvironment. Xu et al. [138] leveraged a multiomic network analysis to identify key metabolic pathways driving gastric adenocarcinoma, highlighting the potential of such integrative approaches in the discovery of novel therapeutic targets. These studies indicate that changes in metabolite concentrations, reflecting changes in energy metabolism, amino acid exchange, and lipid pathways, could be early indicators of carcinogenesis and are essential for risk stratification.

While microbial metabolites, such as SCFAs and polyamines, have been implicated in epigenetic modifications, the extent and specificity of their effects in GC remain uncertain. Conflicting results exist regarding whether certain metabolites are tumor-promoting or protective. This ambiguity may reflect differences in metabolite quantification methods, tissue sampling protocols, and host genetic backgrounds. Few studies employ integrated metabolomic–epigenomic analyses, highlighting a gap in mechanistic understanding [139,140,141].

Microbial metabolites, particularly SCFAs, polyamines, and secondary bile acids, play dual roles in GC by modulating inflammation, epigenetic regulation, and cellular proliferation. Some may exert protumorigenic effects, while others exhibit protective properties. Emerging evidence also supports their potential as non-invasive biomarkers for gastric cancer diagnosis and prognosis, though further validation is needed.

## 5. Oral and Gut Microbiota: Peripheral Players with Central Roles

### 5.1. Impact of Oral Microbiota Translocation to the Stomach

Translocation of oral microbiota into the stomach is a multifactorial process with significant implications for gastric microbial composition and disease pathogenesis. The human oral cavity is home to a complex bacterial community, with hundreds of species of bacteria commonly ingested with saliva. During swallowing, approximately 1.5 × 10^12^ oral bacteria enter the gastrointestinal (GI) tract daily; however, the acidic environment of the stomach substantially decreases their viability, typically reducing the bacterial load by 5-6 orders of magnitude [142]. Therefore, the human stomach is constantly exposed to oral bacteria by swallowing saliva and food, with many species of oral bacteria being detected in the gastric microbiota. Although the highly acidic gastric environment generally inactivates a large proportion of these microbes [28], under altered conditions (such as neutralization of gastric acid, mucosal damage, or changes in the microbial community), a subset of these oral bacteria can survive the hostile environment and colonize the stomach. For example, *Fusobacterium nucleatum* can withstand low pH due to membrane adaptations, such as the synthesis of unique fatty acids via the enzyme FnFabM, which contributes to its survival in the stomach and beyond [143]. In addition, Kitamoto et al. [144] emphasized that oral microbes can translocate to different regions of the GI tract. The authors pointed out that while the indigenous microbiota generally prevent colonization by exogenous bacteria through competitive exclusion and immune mechanisms, disruptions, either through the use of proton pump inhibitors (PPIs) or mucosal injury, can allow for ectopic colonization [144]. Molecular techniques revealed the presence of the DNA of some typical oral species, such as *Actinomyces odontolyticus* and *Rothia dentocariosa*; however, their metabolic activity remained low compared to other species, such as *Tannerella* [28]. These findings suggest that although translocation is common, biological activity in the gastric niche is determined by the physico-chemical state of the stomach.

The translocation mechanism itself is therefore influenced by several factors. The stomach functions as a critical barrier in which approximately 99% of ingested microbes are inactivated; however, when gastric acidity is altered (e.g., when PPIs are used or *H. pylori* infection occurs), the barrier function may be compromised, allowing a higher survival rate of translocated oral bacteria [145,146]. Disturbances of the acidic environment caused by *H. pylori* urease activity may reduce the acidity of the stomach, thus providing an opportunity for oral bacteria to survive and potentially colonize the gastric mucosa [147]. In addition, conditions that reduce gastric acid secretion, such as autoimmune atrophic gastritis or long-term pharmacological suppression by PPIs, tend to alter the composition of the gastric microbiota by facilitating the colonization of oral microbes [148]. Under these conditions, oral bacteria are not only more likely to persist but can also play a role in pathogenic processes. For example, certain oral bacteria are capable of producing carcinogenic compounds, such as acetaldehyde and nitrosamines, which have been implicated in gastric carcinogenesis [42,149]. These observations emphasize that the translocation of the oral microbiota is not just a passive event, as it can actively influence disease outcomes by altering the local microbial ecosystem and possibly interacting with stable gastric pathogens, such as *H. pylori* [43]. In addition, the oral–gastric connection emphasizes the importance of considering the GI tract as a continuous microbial ecosystem rather than an isolated niche. The interaction between the oral and gastric microbiota is increasingly recognized as a potential source of biomarkers for the early detection of gastric diseases, including cancer [150], although current evidence emphasizes primarily microbial co-occurrence and correlation rather than direct causation [42]. Such diagnostic potential is supported by the observed similarity in microbial communities between the tongue and the gastric mucosa in pathologic states, reinforcing the concept of microbial transmission along the digestive tract [43].

Once past the stomach, most oral bacteria continue their journey to the gut, where research on *Porphyromonas gingivalis* indicates that ingested bacteria typically disappear within a few days, showing a significant attrition rate [151]. The acidity-suppressing effects of PPIs lead to increased colonization of resident bacteria in the mouth and the gut, as the reduced acidity creates a more permissive environment for these organisms [152,153]. In addition, the overall health of the intestinal mucosal barrier appears crucial; compromised barriers can facilitate the persistence and expansion of ectopically colonized oral bacteria, contributing to dysbiosis and inflammatory conditions [154,155].

The presence of oral microbiota, particularly *Porphyromonas gingivalis*, in patients with GC is increasingly recognized and appears to occur through several interconnected mechanisms. Evidence suggests that *P. gingivalis*, a key pathogen in periodontal disease, can translocate from the oral cavity into the GI tract, contributing to a proinflammatory and dysbiotic microenvironment that favors tumor progression. Several epidemiologic and pathogenetic studies have linked chronic periodontal infections with adverse outcomes in gastrointestinal cancers, including GC [156,157]. Mechanistically, *P. gingivalis* is well-known for its ability to colonize the oral epithelium and modulate local and systemic immune responses. Producing different virulence factors, the structure and function of mucosal barriers can be altered. This alteration can facilitate its migration into the esophagus and the stomach, thus settling in the gastric mucosa. In the gastric environment, *P. gingivalis* can disrupt local homeostasis through inflammation and immune modulation, processes that are favorable to tumorigenesis [156,158]. For example, experimental models and clinical observations have shown that inflammatory signals induced by *P. gingivalis* can drive carcinogenic processes by increasing cell proliferation, inhibiting apoptosis, and promoting angiogenesis. In addition, significant differences in microbial composition between cancer patients and healthy patients have been reported. Enrichment of oral pathogens, such as *P. gingivalis,* in the gastric microbiome has been observed, suggesting that oral dysbiosis can serve as both a marker and mediator of GC risk [159]. Disruption of the normal gastric microbiota by these oral pathogens can trigger a microenvironment that supports tumor initiation and progression. In addition, the ability of *P. gingivalis* to indirectly modulate host responses implies that its presence in GC patients can not only be incidental but actively contribute to disease severity.

Although growing evidence supports the presence of oral bacteria in the gastric niche and their association with GC, it is important to emphasize that most current studies are correlative. The detection of shared microbial taxa between the oral cavity and the stomach, or even enrichment of oral pathogens, such as *Porphyromonas gingivalis,* in GC patients, does not prove causality [160,161,162]. These overlaps may arise from common environmental factors, impaired mucosal defenses, or changes in gastric pH, rather than direct microbial oncogenesis. Mechanistic studies using animal models and microbial knockouts are still limited, and the biological plausibility of oral-to-gastric tumorigenic progression, although compelling, remains to be conclusively demonstrated. Therefore, while the oral–gastric microbial axis is a promising area for biomarker discovery, caution must be exercised when interpreting these associations as causative [163].

Therefore, oral microbiota can influence gastric microbial composition through translocation and colonization, particularly in patients with poor oral hygiene or periodontal disease. Shared taxa between the oral cavity and the stomach have been associated with inflammatory and carcinogenic processes. However, evidence is largely correlative, and causal pathways remain to be elucidated.

### 5.2. The Salivary Microbiota as a Diagnostic Avenue

The salivary microbiota has emerged as a promising non-invasive biomarker for a variety of diseases due to its ease of collection, stability, and rich molecular content. Recent advances in high-throughput sequencing and salivaomics have enabled detailed characterization of the microbial communities present in saliva, revealing disease-specific signatures that can aid early diagnosis and personalized medicine [164,165]. The inherent advantages of saliva as a diagnostic fluid, such as its non-invasive collection process, minimal patient discomfort, and suitability for repeated or large-scale screening, further support its potential role as a readily accessible biomarker reservoir [164,165]. In addition, the presence of both microbial DNA and RNA in saliva provides information about active microbial communities and host–microbe interactions, as studies using metatranscriptomic approaches have shown [166,167]. The stability of salivary microbiota at different times of the day has also contributed to its usefulness as a biomarker. Salivary bacterial compositions exhibit minimal diurnal variation, thus providing a consistent measure that is essential for reliable disease biomarkers [167]. This temporal stability strengthens the case for the use of salivary microbiota in screening and monitoring chronic conditions, such as periodontal disease, oral squamous cell carcinoma (OSCC), and systemic diseases in which inflammatory processes are key components [166,168]. In OSCC, robust microbial signatures were identified in saliva that distinguish cancer patients from healthy controls and facilitate machine-learning-based predictive modeling for early detection [169]. Similarly, a study evaluating an oral microbiome assay has demonstrated its applicability as a non-invasive diagnostic tool for detecting gastric alterations, offering a new perspective for screening patients undergoing upper gastrointestinal endoscopy [170]. The ability of the salivary microbiome to serve as a biomarker extends to systemic applications, as its composition has been found to correlate with colorectal polyps and inflammatory bowel disease (IBD), linking the oral cavity to gastrointestinal and systemic health outcomes [171,172].

The interaction between salivary microbiota and host physiology extends beyond the oral cavity. Investigations have shown that bacteria in saliva can affect the composition and function of the GI microbiota, thereby influencing systemic health and disease states [173]. This interplay not only supports the potential diagnostic value of salivary biomarkers in GI diseases but also emphasizes the broader implications of the mouth–gut axis in pathogenesis [173]. In addition, studies have demonstrated that changes in salivary microbial communities are associated with immune responses and metabolic changes, providing further evidence of their ability to serve as non-invasive biomarkers reflecting systemic pathological conditions [174,175].

While the diagnostic potential of the salivary microbiota is increasingly supported by emerging evidence, it is important to emphasize that most of the existing studies establish correlative relationships rather than causative mechanisms. The identification of disease-associated microbial signatures in saliva may reflect downstream effects of systemic inflammation or mucosal alterations rather than direct etiological roles in gastric pathogenesis. Moreover, the majority of studies are cross-sectional and lack longitudinal validation or mechanistic investigation [150,173,176,177]. Therefore, although salivary microbiota hold promise as accessible biomarkers for gastric and systemic diseases, their clinical utility must be interpreted with caution, and future work should focus on clarifying whether these microbial patterns are passive indicators or active drivers of disease.

The salivary microbiota represents a promising, non-invasive source for gastric cancer biomarker discovery. Specific bacterial taxa show potential in distinguishing gastric cancer from non-malignant conditions. However, diagnostic accuracy is influenced by host, lifestyle, and methodological factors, highlighting the need for standardization and large-scale validation.

### 5.3. The Digestive Tract Microbiota’s Systemic Immunoregulatory Effects

**Figure 6:**
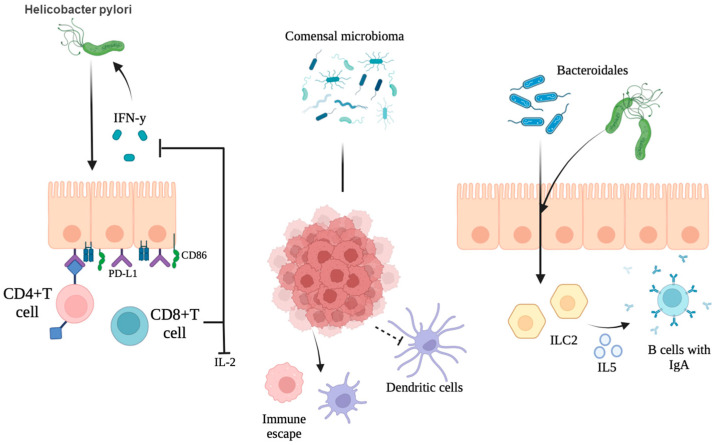
Influence of the gut microbiome on immune regulation in the stomach.

The gut microbiota plays a key role in shaping local and systemic immune responses that extend to the gastric mucosa, forming an integral part of the gut tract–stomach axis. The mechanisms underlying this influence involve multifactorial interactions between microbial metabolites, immune cell modulation, and the maintenance of mucosal barrier integrity. The gut microbiome, comprising trillions of microorganisms, plays a vital role in maturing and modulating the mucosal immune system, which in turn shapes systemic immunity [178,179]. These microorganisms produce several metabolites, particularly SCFAs, that serve as critical signaling molecules to promote the differentiation of regulatory T cells and dampen excessive inflammatory responses [178,180]. Such metabolic products provide feedback that strengthens the integrity of the intestinal barrier while exerting distal effects on immune homeostasis throughout the body [179]. The process of translocation of oral microbes into the gut, as discussed by Bartlett et al. [172], demonstrates that the interaction between oral and gut microbes can influence the immune environment throughout the GI tract, including the stomach. Resident gut microbiota have been reported to modulate immune responses by interacting with local immune populations, such as macrophages and dendritic cells. For example, Ning et al. [181] have described the crosstalk between intestinal microbiota and intestinal macrophages, highlighting how microbe-derived signals orchestrate the production of cytokines and inflammatory mediators. These modulations are essential for maintaining barrier homeostasis and can have effects on the gastric mucosa, particularly when dysbiosis occurs. Such disturbances in microbial composition can lead to altered cytokine environments that can predispose individuals to inflammatory conditions in the stomach (Figure 6).

The integration of microbial signals in different organs emphasizes a broader immunomodulatory influence. Gebrayel et al. [180] analyze the bidirectional communication between the gut microbiota and host immune cells, highlighting how perturbations in the gut microbial community can alter systemic immune responses. This phenomenon underscores the intestinal tract–stomach axis, demonstrating that alterations in gut-derived immune regulation can affect gastric mucosal responses to resident and translocated bacteria. Understanding these interactions is particularly important in the context of gastric pathologies, where an unbalanced immune response, which can originate from microbial dysbiosis, can favor a proinflammatory environment conducive to neoplastic transformation [161]. These studies indicate that the gut microbiota exerts a broad immune influence that is essential in controlling mucosal inflammation and maintaining barrier function along the GI tract. Disturbances of these finely regulated immune processes, both through changes in microbial composition and loss of immunoregulatory metabolites, can compromise the integrity of the gastric mucosa and contribute to the pathogenesis of the disease.

Pathogen-associated molecular patterns (PAMPs) derived from commensal bacteria are recognized by pathogen recognition receptors (PRRs) on intestinal epithelial cells and antigen-presenting resident cells, thereby establishing a basal level of tonic stimulation essential for immune surveillance and tolerance [179,182]. This basic activation ensures that the immune system can quickly mobilize against pathogens while avoiding over-reactivity to harmful antigens in food or commensals. In addition, the interaction between microbiome-derived metabolites and host-encoded receptors modulates intracellular signaling cascades, such as the JAK2/STAT3 pathway, thereby regulating neutrophil responses and consequently influencing systemic inflammatory states [183,184].

**Figure 7:**
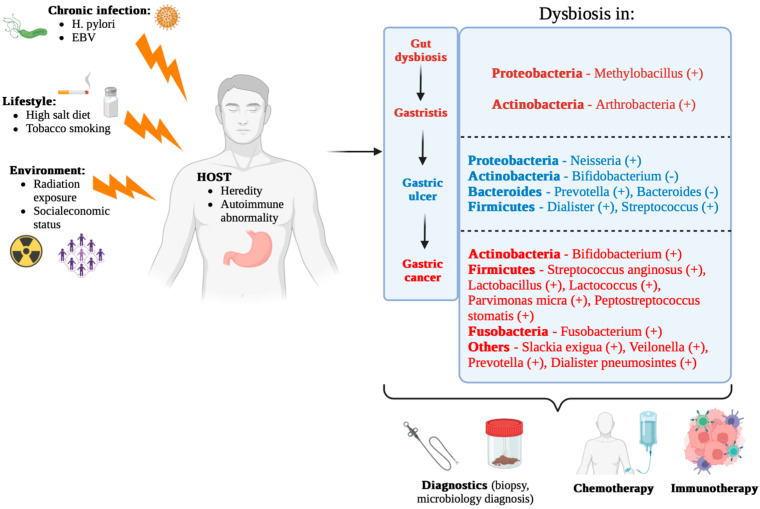
Microbiome dysbiosis in GC.

In addition to maintaining homeostasis, the gut microbiota also modulates antitumor immunity by reprogramming local and systemic immune microenvironments. Dysbiosis or disruption of the gut microbial community can change this delicate equilibrium, creating a permissive environment for tumors characterized by diminished immunosurveillance and altered cytokine profiles [185]. This change not only directly affects the tumor microenvironment but also influences the efficacy and side effect profile of cancer therapies [185]. Such insights have fueled interest in therapeutic strategies that exploit changes in the gut microbiota to enhance antitumoral immune responses and improve treatment outcomes. In addition, gut dysbiosis has been implicated in the pathogenesis of immune-mediated intestinal diseases and other systemic inflammatory disorders. The dynamic interaction between the gut microbiota and the mucosal immune system is essential for the regulation of inflammatory processes, as evidenced by the ability of microbial communities to influence immunoregulatory metabolites, cytokine production, and leukocyte function [178,186]. These observations underline the potential of modulation of the targeted microbiota as a means of restoring immune balance and alleviating chronic inflammatory conditions (Figure 7). 

Changes in the oral microbial community (oral dysbiosis) can contribute to the development of gastric neoplasia through several interlinked mechanisms. Oral dysbiosis, characterized by changes in the composition and relative abundance of commensal and pathogenic species, can lead to the translocation of bacteria from the oral cavity into the stomach. Several studies have shown that in the presence of factors like decreased gastric acidity or *H. pylori* infection, oral bacteria can colonize the gastric mucosa, thereby establishing a dysbiotic microenvironment that predisposes to malignant transformation [43,187].

Wu et al. [43] reported a significant co-occurrence between bacteria present in the oral cavity and gastric mucosa in *H. pylori*-infected individuals. This finding indicates that the oral microbiota can act as a reservoir of potential pathogens that directly influence the composition of the gastric microbiome. Consistent with this statement, You et al. [187] found that correlations between the oral and gastric microbiomes become more pronounced during the progression of gastric carcinogenesis, from early mucosal changes to cancer manifestation, implying that ectopic colonization by oral taxa could contribute to the neoplastic process. In addition, culture-independent high-throughput sequencing showed that the gastric microbiota in patients with gastric neoplasia is distinctly different from that of non-cancer patients. Hua et al. [188] observed that patients at risk of GC have an increased abundance of bacterial species of oral origin, which can promote inflammatory conditions favorable to carcinogenesis. Similarly, Ferreira et al. [82] demonstrated that gastric carcinoma is associated with a reduced abundance of *Helicobacter* and an enrichment of other bacterial genera, many of which are recognized as oral commensals, suggesting that a shift towards an oral-type microbiome in the stomach could facilitate the formation of a nitrosating microbial community involved in the production of carcinogenic compounds.

Oral dysbiosis can lead to gastric neoplasia through multiple mechanisms. Sun et al. [160] discussed how oral bacteria contribute to cancer evolution and progression through pathways that include chronic inflammation, immunosuppression, antiapoptotic activity, and the production of carcinogens. Bakhti and Latifi-Navid [189] pointed out that the oral microbiota plays a role in both modulating gastric homeostasis and persistent colonization of the gastric mucosa, and, in the context of altered host defense and microenvironmental changes, it can facilitate progression to malignant lesions.

## 6. Microbiota, Immunity, and Therapy Response

The gastric tumor microenvironment (TME) is a complex and dynamic entity composed not only of neoplastic cells but also diverse populations of stromal and immune cells. Among these, tumor-associated macrophages (TAMs), myeloid-derived suppressor cells (MDSCs), lymphocytes, endothelial cells, and fibroblasts interact in a complex network that ultimately dictates tumor progression and therapeutic response [190,191]. In this complex environment, cellular and non-cellular components, including factors secreted by commensal and intratumoral microbiota, play a crucial role in shaping the immune landscape of gastric cancer.

The microbiota exerts profound immunomodulatory effects in the gastric TME. *Streptococcus mitis*, identified in tumor tissues, has been shown to inhibit GC progression by suppressing M2 macrophage polarization, a phenotype commonly associated with immune suppression and tumor promotion [192]. In contrast, *Akkermansia muciniphila*, a commensal bacterium of the gut, has demonstrated the ability to promote M1 macrophage polarization and enhance cytotoxic immune responses, thus potentially enhancing the efficacy of therapeutic strategies [193]. These findings highlight that specific microbial constituents can differentially modulate macrophage subtypes, influencing the balance between protumor and antitumor immune responses in the gastric TME.

Changes in gastric microbiota composition have been strongly correlated with changes in immune cell infiltration and function. Dysbiosis in the gastric mucosal environment has been associated with an increase in suppressive immune cells, such as regulatory T cells (Tregs) and plasmacytoid dendritic cells (pDCs), which contribute to immune evasion and facilitate tumor progression [194]. Such dysbiotic states can also modulate cytokine profiles and immune gene signatures in tumors, thereby affecting immunosurveillance and the overall efficacy of immune responses [195]. Enrichment or depletion of certain microbes in tumor tissues appears to program the immune microenvironment to a stimulatory or suppressive state, influencing clinical outcomes.

### 6.1. Impact of Microbiota on Immunotherapy Outcomes

Certain microbial species and their metabolites critically influence both the regulation of immune checkpoints and T cell responses, thus affecting the effectiveness of cancer immunotherapy. Several studies have demonstrated that the gut microbiota contributes to the modulation of immune checkpoints, such as PD-1/PD-L1 and CTLA-4, through a combination of direct effects on T cell differentiation and indirect modulation of antigen presentation and cytokine production [196,197]. Certain commensal bacteria produce metabolites (SCFAs and LPS), which have been shown to modulate the local immune microenvironment. SCFAs have been implicated in the consolidation of the antitumor immune response by modulating gene expression in T cells and promoting a regulatory balance in the TME [196,198]. LPS can activate innate immune pathways that ultimately shape adaptive T cell responses, influencing the effectiveness of immune checkpoint inhibitors (ICIs) [196,199].

Certain microbial taxa can directly regulate the composition and function of T cell repertoires. Commensal species have been reported to influence the differentiation of CD4+ T cells into Tregs and T helper 17 (Th17) cells in an antigen-dependent manner, thereby modulating adaptive immune responses [200,201]. This balance between Tregs and Th17 cells is crucial, as an appropriate ratio is needed to prevent tumor-driven immune suppression while avoiding exaggerated inflammatory responses. Some bacteria are known to induce Treg cells, contributing to immune tolerance, while others promote Th17 differentiation, thus potentially enhancing antitumor immunity [200,201].

Regulation of ICI by the microbiota also involves modulation of antigen presentation mechanisms. Intestinal epithelial cells (IECs) express major histocompatibility complex (MHC) class II molecules at the host–microbiota interface, a process that is integral to directing microbiota-specific T cell responses [202]. This interaction illustrates a critical pathway through which microbial signals can fine-tune the adaptive immune response, influencing how T cells respond to tumor antigens and, in turn, affecting the response to ICI therapy [202,203].

The gut microbiota has emerged as a key factor influencing the clinical outcomes of cancer immunotherapy, particularly in ICIs targeting the PD-1/PD-L1 axis. Several clinical and preclinical studies demonstrate that gut microbiome composition and diversity can modulate therapeutic efficacy. For example, antibiotic exposure, which disrupts the native microbial community, has been associated with poorer survival outcomes in patients receiving PD-1 inhibitors [204]. These observations suggest that certain microbial signatures can serve as predictive biomarkers for response to immunotherapy, emphasizing the need to understand pathogenic links between microbiota composition and immune modulation in cancer therapy [199,204].

The gut microbiota helps modulate immunity by producing metabolites and modulating local cytokine environments that enhance antitumor immunity. Specific bacterial species and their metabolic products have been implicated in enhancing the antitumor effects of ICI; for example, alterations in the gut microbiome can modulate the balance between regulatory and effector T cells, ultimately affecting T cell activation and proliferation [196,205]. Distinct microbial communities enriched with beneficial species (e.g., *Akkermansia muciniphila* and members of the *Ruminococcaceae* family) have been correlated with improved responses to PD-1 blockade, suggesting that microbial metabolites can enhance immune cell cytotoxicity [206]. These findings support an emerging therapeutic paradigm in which modulation of the gut microbiota through probiotics or fecal microbiota transplantation can be used as an adjunct to immunotherapy to improve patient outcomes [196,207].

The efficacy of immunotherapy by blocking PD-1/PD-L1 is substantially influenced by the composition and functionality of the gut microbiota. Several studies emphasize that the gut microbiome can act as a key regulator by modulating both innate and adaptive immune responses and thereby affecting the clinical outcomes of PD-1/PD-L1 blockade therapy [208,209,210]. It has been emphasized that favorable gut microbial communities, including the presence of beneficial commensals, contribute to an enhanced antitumor immune response, which may enhance the efficacy of ICIs [208,209]. Various mechanisms through which the intestinal microbiota influences the TME and potentially enhances the efficacy of PD-1/PD-L1 blockade have been reported [199,210]. The basic mechanisms involve the modulation of antigen-presenting cell function, the activation of T cells, and the balance between effector and regulatory T cells. *Bifidobacterium species* have been identified as key bacteria in this process. Sivan et al. [211] demonstrated that oral administration of *Bifidobacteria* could improve dendritic cell function and enhance priming of CD8+ T cells within tumors, leading to improved responses to PD-L1 blockade. These findings are supported by the study by Frąk et al. [212], who highlighted that changes in specific bacterial populations can recalibrate immune responses, promoting antitumor immunity and attenuating inflammatory toxicity. Such modulation affects not only local immune contexts but also systemic immune surveillance critical for sustaining the effectiveness of checkpoint inhibition.

Emerging evidence indicates that the gut microbiota not only primes the host immune system but also responds dynamically to immunotherapeutic interventions. In breast cancer models, Buchta Rosean et al. [213] showed that commensal dysbiosis (a loss of microbial homeostasis) negatively affects the efficacy of PD-L1 blockade, highlighting the importance of maintaining a balanced microbiome for optimal therapeutic performance. In addition, Kim et al. [214] showed that restoration of a healthy microbial composition by FMT can overcome resistance to anti-PD-1 inhibitors in advanced solid cancers, especially gastrointestinal cancers. Furthermore, Zou et al. [215] further strengthen these perspectives by discussing combination therapies that target both the tumor and its associated microbial environment, paving the way for a more personalized and effective approach to cancer immunotherapy. These interconnected studies suggest the potential for integrating microbiota-modifying strategies as an adjuvant to PD-1/PD-L1 blockade therapy. By harnessing the influence of the gut microbiota on immune homeostasis and antitumor responses, clinicians can enhance the efficacy of current immunotherapies in different types of cancer.

A particularly intriguing aspect of microbiota-driven immune modulation involves its impact on tissue-resident memory T cells (TRM), particularly CD8+ TRM, which are essential for localized tumor immunosurveillance. CD8+ TRM CD8+ have been shown to infiltrate the tumor microenvironment and are associated with robust immunostimulatory responses that potentiate the efficacy of ICIs. Local activation and persistence of CD8+ TRM appear to be modulated by signals derived from microbial metabolites, with evidence indicating a positive correlation between certain commensal bacteria and the abundance as well as the functional status of these cells [216,217]. For example, experimental data have shown that enrichment of CD8+ TRM within tumor sites is linked to improved responses to PD-1 inhibitors, and those microbial indicators are essential for the maintenance and activation of these cells [217]. Furthermore, the interaction between gut microbiota and CD8+ TRM CD8+ is increasingly recognized as a potential target to overcome immunotherapy resistance. Altered microbial communities can disrupt the balance between immunostimulatory and immunosuppressive signals within the tumor microenvironment, thereby affecting the expansion and cytolytic potential of CD8+ MTR CD8+ [218]. Clinical data underscore this notion, indicating that patients with gut microbiota profiles that favor TRM activation exhibit stronger antitumor responses and prolonged survival following ICI treatment [216,219]. Therefore, these studies highlight the importance of considering both systemic and tissue-specific immune modulation, driven by the microbiota, when designing and optimizing immunotherapeutic strategies.

### 6.2. Strategies to Modulate Microbiota to Enhance Therapeutic Response

Modulating the gut microbiota to improve therapeutic outcomes, particularly in the context of immunotherapy, has emerged as a promising strategy that exploits the complex interaction between host immunity and microbial communities [220,221]. Specific approaches, such as probiotic supplementation, fecal microbiota transplantation (FMT), dietary modifications, and targeted administration of microbial metabolites, can shift the TME from an immunosuppressive to an immunostimulatory state, thereby potentiating the efficacy of ICI [195,222]. In preclinical models and early clinical investigations, administration of commensal bacteria (such as *Bifidobacterium* and *Lactobacillus* spp.) has been associated with increased CD8+ T cell infiltration and improved responses to immunotherapy, highlighting the value of probiotic strategies in remodeling immune profiles within tumors [220,223].

Gut-derived microbial metabolites (e.g., SCFAs) influence the differentiation and function of effector T cells and regulatory T cells, thereby altering cytokine production and immune cell trafficking within the TME [221,224]. These metabolites can also modulate systemic inflammation and support the maintenance of tissue-resident memory T cells, which are essential for robust antitumor immunity [224]. Beyond providing beneficial metabolites, FMT (the process through which the microbiota of a healthy donor is transferred to a patient with dysbiosis) has been shown to restore a favorable microbial composition, thereby enhancing the therapeutic impact of ICI by promoting immune cell activation and reducing protumor inflammation [195,221].

Nutritional therapies offer a promising approach to modulate gut microbiota and potentially mitigate gastric cancer progression. Nutritional approaches, including the adoption of prebiotic fiber-enriched diets or the Mediterranean diet, polyphenols, and fermented foods, can enhance the growth of beneficial bacteria, such as *Lactobacillus* and *Bifidobacterium*. These beneficial microbes produce SCFAs, which have anti-inflammatory properties and can consolidate the intestinal barrier, reducing the risk of carcinogenesis [225]. This nutritional modulation not only improves intestinal barrier function and the regulation of systemic immunity but also synergizes with immunotherapeutic agents by creating an environment conducive to enhanced antitumor responses [225]. In addition, emerging strategies have explored the use of modified probiotics and microbial metabolite cocktails to selectively induce immunogenic cell death in tumors, further transforming “cold” tumors into “hot” tumors that are more easily blocked by checkpoints [226].

Probiotic and prebiotic interventions are also being explored as adjuvant therapies in the treatment of CG. Probiotics can help restore the microbial balance and improve the immune response, while prebiotics provide the substrates necessary for beneficial bacteria to thrive. Combining these nutritional strategies with conventional cancer therapies can increase the effectiveness of treatment and reduce side effects [227].

**Figure 8:**
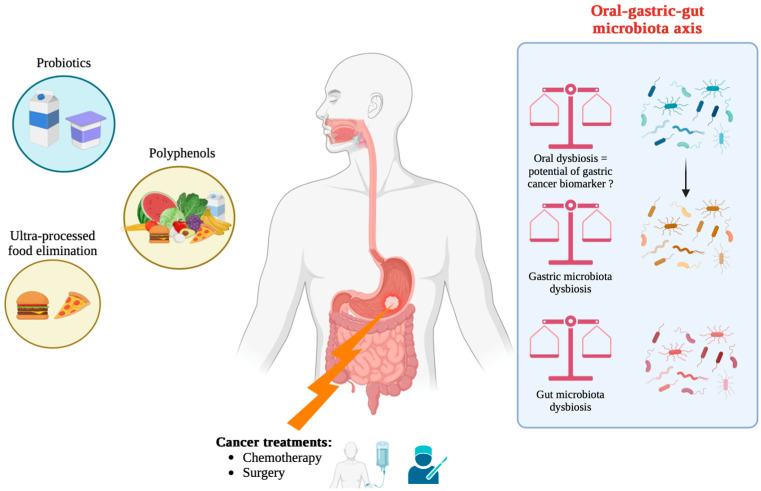
Potential nutritional interventions that can positively influence the oral–gastric–intestinal microbiota during GC treatment.

Modulating the gut microbiota through probiotics, FMT, dietary modifications, and microbial metabolite supplementation can reprogram the TME and potentiate the efficacy of immunotherapy. These approaches capitalize on the dual role of the microbiome as both a biomarker and a therapeutic target, offering new and compelling ways to overcome ICI resistance and improve patient outcomes in cancer therapy [195,220,221,222,223,224,225,226]. Continued research is crucial to fully understand both the interaction between the microbiota and GC and to optimize dietary interventions for gastric cancer patients (Figure 8) [227].

## 7. Clinical Perspectives and Future Directions

### 7.1. Clinical Aspects

In a biological setting, oral dysbiosis contributes to a change in the gastric microbiome. This change creates a microenvironment favorable to chronic inflammation and the accumulation of genotoxic compounds, which together potentiate carcinogenic processes in the stomach. Therefore, the integration of molecular, microbiologic, and clinical data from investigations reported in the literature supports the idea that monitoring and possible microbiota modulation can have preventive and/or therapeutic implications for gastric neoplasia [43,82,187,188,189].

**Figure 9:**
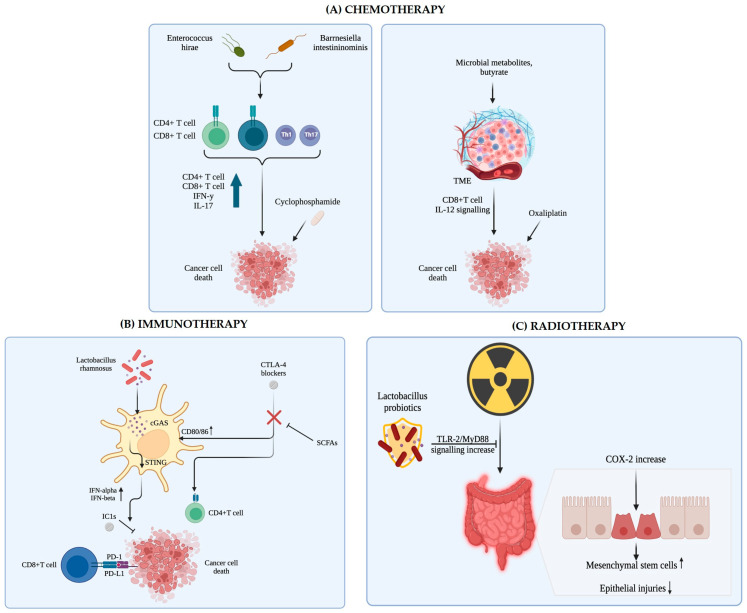
Mechanisms of the microbiota’s impact on cancer treatment effectiveness.

The gut microbiota plays a key role in modulating the efficacy and toxicity of anti-cancer therapies. Emerging evidence highlights how the gut microbiota influences the response to chemotherapy, immunotherapy, and other cancer treatments through various molecular mechanisms (Figure 9) [228].

Studies in several disease models have shown that alterations in microbial composition and function correlate with disease severity, progression, and response to treatment, paving the way for precision medicine approaches. For example, in systemic sclerosis, distinctive fecal microbiome profiles have been associated with gastrointestinal complications that undermine patients’ quality of life. Fiorentini et al. [229] highlighted that assessing the microbiota early in the course of the disease can help stratify risk and identify candidates for targeted microbial therapies to manage small intestinal bacterial overgrowth. Similarly, in metabolic disorders, such as type 2 diabetes and nonalcoholic fatty liver disease (NAFLD), changes in the gut microbial community have been associated with clinical indices to predict disease progression and guide metabolic therapies [230,231]. Mishra et al. [232] further emphasize that gut microbiota could serve as prognostic markers in metabolic diseases by modulating responses to diet and drugs.

Inflammatory and autoimmune diseases support the role of microbiota as biomarkers. Multiomics studies demonstrate that microbial signatures derived from metagenomic analysis in inflammatory bowel diseases can be used to assess disease activity and predict complications [233]. This concept is also extended to systemic lupus erythematosus and inflammatory bowel disease, respectively, where specific microbial patterns offer prognostic and therapeutic stratification potential [234,235]. Prognostic utility is evident beyond chronic inflammatory diseases. In pulmonary hypertension, Yang et al. [236] reported that microbial metabolites possess significant clinical value, suggesting that these compounds could act as surrogate markers for disease severity and treatment efficacy.

The scope of microbiota-based biomarkers is particularly profound in oncology. It has been shown that gut microbial composition can influence tumor progression and mediate responses to immunotherapies in cancers like pancreatic cancer and non-small cell lung cancer [237,238]. In a broader context, it discusses how dysbiotic profiles serve as non-invasive diagnostic tools and inform therapeutic decisions by correlating specific microbiota signatures with clinical outcomes [239,240]. Xu et al. [241] and Maccauro et al. [242] corroborate this association in hematological and cholangiopathic disorders by correlating specific bacterial taxa with prognostic indices and therapeutic criteria.

In infectious diseases, especially COVID-19 and sepsis, several studies highlight the relevance of changes in the gut microbiota as biomarkers for disease severity and treatment response, highlighting that changes in microbial community composition, including commensal and opportunistic pathogen variations, can predict clinical outcomes and thus serve to stratify patients for personalized therapeutic regimens [243,244,245,246].

Therefore, in different clinical contexts—from metabolic and autoimmune diseases to cancer and acute infections—the gut microbiome emerges as a promising reservoir of non-invasive biomarkers. These biomarkers facilitate early diagnosis and risk stratification and have the potential to inform and refine therapeutic interventions.

The salivary microbiota has emerged as a promising non-invasive diagnostic pathway that exploits the wealth of microbial and molecular information contained in saliva. The concept is based on the observation that saliva, a complex biofluid containing bacteria, human DNA, RNAs (including microRNAs), proteins, and metabolites, reflects both local oral health and systemic physiological states. Unlike invasive tissue biopsies or blood sampling, saliva sampling is simple, safe, and cost-effective [247]. Detailed microbial profiling using advanced next-generation sequencing and microfluidics has shown that the composition of the salivary microbiome can provide essential information on various diseases. Correlations have been demonstrated between bacterial communities in saliva and those in subgingival plaque, emphasizing its potential to reflect periodontal status as well as broader host–microbe interactions [248]. Integrating microbial profiles with other salivary constituents, including proteins, metabolites, and nucleic acids, could increase the sensitivity and specificity of diagnostic tools. The multi-omics approach in salivary biomarker research paves the way towards precision medicine, allowing clinicians to assess disease risk, monitor progression, and predict therapeutic responses in real time [164,249]. Advances in salivary miRNA testing complement microbial measurements, enabling rapid ‘sample-to-response’ diagnostic platforms that have potential application across a spectrum of diseases, including cancers and neurodegenerative disorders [250]. Continued refinement of sequencing technologies and bioinformatics analyses, in addition to longitudinal studies to establish causal relationships, will be essential for the validation and standardization of saliva-based biomarkers for clinical use. The integration of high-throughput sequencing, bioinformatics, and machine learning in salivary biomarker analysis could revolutionize early disease detection, personalized medicine, and routine clinical screening while minimizing patient discomfort and reducing healthcare costs [170,250,251]. This non-invasive approach offers substantial promise as a diagnostic pathway that bridges microbiome research with broader clinical applications.

From our point of view, we consider that precision medicine approaches now incorporate microbial biomarkers alongside genomic and proteomic data to stratify patients by (i) risk of cancer progression (e.g., presence of *Fusobacterium*, low *Lactobacillus*), (ii) likelihood of ICI response (e.g., gut *Akkermansia* enrichment), and (iii) metabolomic profiles associated with immune modulation or chemoresistance.

In the future, personalized microbial signatures may guide preventive interventions (diet, probiotics), treatment optimization (FMT donor selection, microbiome editing), and toxicity mitigation strategies (e.g., immunotherapy-associated colitis).

From a regulatory standpoint, authorities like the Food and Drug Administration (FDA) and the European Medicines Agency (EMA) have begun defining frameworks for investigational microbiota therapeutics. However, standardization of microbial therapeutics, long-term safety data, and ethical issues around donor use remain unresolved challenges.

### 7.2. Future Perspectives

Clinical applications of microbiota modulation are being explored to improve cancer therapy outcomes. Strategies include the use of probiotics, prebiotics, and fecal microbiota transplantation to restore a healthy balance of the gut microbiota. These interventions aim to increase therapeutic effectiveness, reduce treatment-related side effects, and improve overall patient outcomes. Microbial profiling and personalized microbiota-targeted therapies are promising approaches in oncology. Understanding the composition of a patient’s microbiota can help tailor treatment strategies to optimize therapeutic responses and minimize adverse effects [228]. Understanding changes in the gastric microbiota and its metabolites opens new avenues for potential diagnostic biomarkers and therapeutic targets. Early detection and modulation of microbial dysbiosis could play a crucial role in preventing the progression of precancerous conditions to neoplasm.

Several therapeutic strategies are being explored to restore the microbial balance and reduce cancer risk.

Probiotics and prebiotics: These interventions aim to promote the growth of beneficial bacteria and restore microbial balance. Probiotics, live beneficial bacteria, can improve gut health and inhibit the growth of pathogenic bacteria. Prebiotics, non-digestible food components that promote the growth of beneficial bacteria, can also contribute to microbial balance. The human stomach, traditionally considered too hostile to microbial life because of its acidic environment, is home to a diverse microbiota that plays crucial roles in health and disease [252].

Gastric microbiota composition is influenced by factors like diet, age, and *Helicobacter pylori* infection. *H. pylori*, a well-known pathogen, is a major contributor to chronic gastritis, peptic ulcers, and gastric cancer. Its presence disrupts the gastric microbial balance, leading to dysbiosis, which exacerbates inflammation and disease progression.

Probiotics, living microorganisms that confer health benefits to the host, have emerged as promising therapeutic agents for restoring microbial balance and treating gastroduodenal diseases. Specific probiotic strains, such as *Lactobacillus* and *Bifidobacterium*, can inhibit *H. pylori* colonization, improve mucosal barrier function, and modulate immune responses. Probiotics can reduce *H. pylori* density, improve eradication rates when combined with antibiotics, and mitigate the side effects of antibiotic therapy [211,253,254]. In addition, probiotics have anti-inflammatory properties, which are beneficial in managing conditions like gastritis and peptic ulcers. They can increase the production of anti-inflammatory cytokines and reduce the expression of proinflammatory mediators, thereby promoting mucosal healing and relieving symptoms.

In conclusion, the stomach microbiota plays a vital role in gastroduodenal health, and probiotics provide a valuable tool for managing associated diseases. Ongoing research is essential to optimize probiotic formulations and determine the most effective strains and doses for specific conditions [252].

Antibiotic and antifungal therapies: Targeting specific pathogens with antibiotics and antifungals can help reduce microbial dysbiosis. However, the use of these therapies needs to be carefully managed to avoid disruption of the beneficial microbiota and to prevent the development of antibiotic resistance.

Dietary changes: Diet plays a significant role in shaping the gut microbiota. Environmental and lifestyle factors, including diet, physical activity, stress, and the use of antibiotics or other drugs, continually reshape the gut’s microbial landscape [20,255,256]. Diets high in dietary fiber support a diverse and balanced microbiota, while highly processed and low-fiber diets contribute to dysbiotic conditions that can promote inflammation [255,256].

Dietary intake of polyphenol-rich foods has been associated with a lower incidence of GC, highlighting the potential of dietary interventions in cancer prevention. Polyphenols, natural compounds found in fruits, vegetables, tea, and wine, have attracted significant attention for their anti-cancer properties, particularly against GC [257]. Polyphenols fight GC via several molecular pathways. They exhibit powerful antioxidant properties, neutralizing free radicals and reducing oxidative stress, a key factor in carcinogenesis. In addition, polyphenols can inhibit cell proliferation and induce apoptosis in cancer cells. Moreover, polyphenols possess anti-inflammatory properties that help to alleviate chronic inflammation, a known risk factor for GC. They inhibit the production of proinflammatory cytokines and enzymes, such as COX-2, thereby reducing inflammation and associated damage to gastric tissues [257]. The interaction between polyphenols and gut microbiota further enhances their anticarcinogenic effects. Gut microbiota can metabolize polyphenols into bioactive compounds with increased bioavailability and potency (e.g., conversion of ellagitannins to urolithins by gut bacteria).

Fecal microbiota transplantation: FMT can have a role in treating GC patients in the future by modulating the composition of the gut flora. However, further studies are needed to assess the safety of this procedure and to mitigate the risk of side effects [258,259].

Bacteriophage-based strategies: Bacteriophages are viruses that selectively infect bacteria, and are more than a hundred times more common in the gut than in bacteria and also in human cells [260]. Bacteriophages have been studied for the treatment of colon cancer, targeting pathogenic bacteria without interfering with normal microbiota [261]. As for gastric tumors, recent research on phage strategies targeting *H. pylori* can open new doors for treating patients with *H. pylori*-positive GC [262].

Bile acid modulation: Given the role of bile acids in microbial dysbiosis, therapies that modulate bile acid levels could help restore microbial balance. This could involve dietary changes, drugs, or microbiome-targeted therapies. Understanding the interplay between bacterial bile acid metabolism and DNA damage highlights potential therapeutic and preventive strategies. Interventions aimed at modulating the gut microbiome or directly altering the composition of bile acids in the stomach could attenuate the generation of ROS and subsequent DNA damage, ultimately reducing the risk of gas-targeted carcinogenesis [106,107]. Evidence reported in the literature emphasizes the importance of secondary bile acids in the pathogenesis of GC, suggesting that microbial modulation and bile-acid-targeted therapies are promising in the prevention and treatment of this malignancy [106,109,110,111].

Immunotherapy and personalized medicine: Advances in immunotherapy, particularly ICI, offer promising avenues to treat GC by harnessing the body’s immune system. The gut microbiota exerts a dual influence on immunotherapy outcomes by modulating not only systemic immune responses to ICI but also local activation of tissue-resident memory CD8+ T cells in the TME. The synergistic interaction between specific microbial constituents and immune effector cells highlights a promising frontier for increasing ICI efficacy and overcoming therapeutic resistance. Therefore, incorporating microbiota-targeted interventions can represent a novel and compelling strategy to improve the clinical management of cancer patients undergoing immunotherapy [216,218,219]. Personalized medicine approaches that take into account a person’s microbiota and genetic profile can optimize treatment strategies and improve outcomes. The systemic immunoregulatory effects of the gut microbiota are mediated by a complex network of microbiota-derived signals, host-receptor-mediated pathways, and metabolite-driven modulation of immune cell functions. As these interactions are understood, therapeutic opportunities aimed at correcting dysbiosis and enhancing beneficial host–microbe interactions hold promise for the management of a wide range of immune-related disorders, including cancer and chronic inflammatory diseases [174,178,179,183].

Microbial interactions with the TME can either suppress or facilitate immune surveillance, directly influencing disease progression and therapeutic response. From a translational standpoint, microbiota-informed strategies, such as probiotics, FMT, and postbiotics, hold promise but require further validation in robust, controlled clinical trials. The integration of microbiome and metabolome profiling into precision oncology platforms is an emerging frontier that could enable early detection, risk stratification, and personalized treatment strategies. In conclusion, strategies aimed at modulating the gut microbiome, such as dietary interventions, probiotics, and fecal microbiota transplantation, offer promising avenues to improve the efficacy of immune checkpoint blockade in cancer therapy [196,197,263,264].

### 7.3. Microbiome-Based Clinical Trials

Recently updated clinical practice guidelines highlight the changing landscape in GC management. European Society of Medical Oncology (ESMO) Clinical Practice Guidelines [265] and guidelines of the Chinese Society of Clinical Oncology (CSCO) [266] highlight new recommendations incorporating immunotherapy, targeted therapies, and anti-angiogenic treatments in advanced and metastatic stages. These guidelines also emphasize updated screening protocols, including hereditary screening, and refined stratifications based on molecular markers, such as HER2 status, mismatch repair deficiency, and microsatellite instability-high (MSI-H) [265,266].

Table 1 presents representative examples of clinical studies regarding the involvement of the microbiota in GC, classified by study design, biological material used, objective, and principal findings. The selected studies reflect recent advances published in the last decade, emphasizing translational relevance and methodological diversity. This comparative overview facilitates a better understanding of microbial dynamics at different stages of gastric carcinogenesis and may guide future research directions.

Immunotherapy remains at the forefront of research and clinical application. Recent randomized trials (such as ATTRACTION-4 and neoadjuvant trials) have demonstrated the benefits of combining ICI with conventional chemotherapy regimens [275,276]. Such combinations showed improvements in progression-free survival and overall survival, particularly in HER2-negative and MSI-H populations [275,277]. In addition, real-world investigations have highlighted the challenge of inter-individual variability, prompting continued research on predictive biomarkers. Studies have identified potential predictors, including PD-L1 expression and PD-1+ circulating memory T cells [278,279]. Complementary analyses using advanced computational methods, deep AI algorithms, and pathomics-based ensemble models (digital anatomical pathology models) have further refined patient stratification and treatment personalization [280,281].

Exploration of the tumor microenvironment has also received increased attention. The role of tertiary lymphoid structures and tumor-infiltrating B cells has been linked to improved immunotherapeutic responses [282], while circulating biomarkers, such as the neutrophil/lymphocyte ratio and indices of systemic immunoinflammation, have emerged as accessible tools for predicting immunotherapy’s efficacy [283]. In parallel, studies investigating the impact of the gut microbiome have revealed its potential role in modulating treatment response in advanced HER2-negative GC [284], suggesting that host–microbial interactions may influence therapeutic outcomes.

Innovations in targeted therapies have also enriched the therapeutic arsenal. In cases where heterogeneity has hindered progression, anti-angiogenic drugs, such as Apatinib, sometimes used in combination with agents like S-1, offer alternative strategies to overcome tumor heterogeneity [285]. In addition, ongoing efforts to use immune checkpoint inhibitors in the neoadjuvant setting offer promising avenues for improving surgical outcomes and long-term survival in resectable gastric and gastroesophageal junction tumors [276,286].

Finally, the integration of multimodal therapeutic approaches, combining the benefits of surgery, chemo-radiotherapy, and immunomodulatory strategies, is still being applied [287]. Such integrated treatment regimens are poised to address the complex biology of gastric cancer by harnessing a combination of therapeutic modalities tailored to individual patient profiles. These developments illustrate a comprehensive shift from conventional monotherapies to personalized multimodal treatment strategies in CG. These advances are underpinned by advances in technology, the discovery of novel biomarkers, and international consensus on best practice, all aimed at improving patient outcomes in this challenging spectrum of diseases.

However, despite significant advances in microbiota research related to gastric carcinogenesis, current studies face important limitations. A substantial number of investigations remain observational and cross-sectional, making it difficult to establish causality between microbial shifts and tumor progression. Additionally, differences in sequencing technologies, sample handling, and taxonomic resolution lead to inconsistent findings across studies and populations. Functional interpretation of microbiota data is often limited, as many studies do not integrate metabolomic or transcriptomic analysis. Moreover, the translatability of preclinical data to human clinical settings remains uncertain due to interspecific differences and simplified experimental models.

## 8. Conclusions

Gastric carcinogenesis is increasingly recognized as a multifactorial process involving intricate interactions between microbial communities, host immune responses, genetic susceptibility, and environmental exposures. While *Helicobacter pylori* remains the most studied pathogen in this context, recent findings have demonstrated that microbial dysbiosis plays an important role in driving the inflammatory and neoplastic processes within the gastric mucosa. The evidence indicates that *H. pylori* not only acts as a direct carcinogen but also reshapes the gastric microbiota by altering pH levels, immune signaling, and nutrient availability. This dysregulation facilitates colonization by opportunistic microbes and reduces the abundance of protective commensal species. Furthermore, fungal elements, such as *Candida albicans,* and translocated oral microbiota like *Fusobacterium* and *Porphyromonas gingivalis* can aggravate mucosal damage and amplify chronic inflammation, accelerating the progression towards malignancy.

Microbial metabolites, including short-chain fatty acids, lactate, and bile acid derivatives, modulate host cell signaling, immune cell function, and epigenetic regulation, either enhancing tumor-suppressive pathways or contributing to oncogenesis. The presence of these metabolites in tumor microenvironments underlines their diagnostic and therapeutic significance.

In the context of cancer therapy, the gastric and gut microbiota have emerged as modulators of the response to immunotherapies, particularly immune checkpoint inhibitors. Specific bacterial taxa, such as *Akkermansia muciniphila* and *Bifidobacterium* spp., have shown potential in enhancing antitumor immunity, while microbial dysbiosis can impair immune surveillance and promote resistance to therapy.

Given these insights, the modulation of the gastric microbiome holds promise as a preventive and adjunctive strategy in GC. Approaches, such as probiotic supplementation, prebiotic dietary modulation, fecal microbiota transplantation, and metabolite-targeted interventions, can offer therapeutic benefits. However, clinical validation remains limited and requires well-designed, controlled trials. Future directions for research include the following:

- Longitudinal studies tracking microbiota changes from precancerous lesions to advanced GC to establish causal relationships and microbial signatures predictive of progression;
- Functional microbiome analyses (metatranscriptomics, metabolomics) to elucidate the active metabolic pathways driving carcinogenesis;
- Identification of microbial-derived biomarkers for early diagnosis, prognosis, and treatment stratification;
- Integration of microbiome data into personalized medicine frameworks, particularly for immunotherapy responsiveness prediction;
- Investigation of microbial–host immune interactions, including the role of microbiota in programming tissue-resident memory T cells and shaping the tumor immune landscape.

Altogether, the gastric microbiota represents a dynamic and clinically actionable component in the pathogenesis and treatment of GC. Continued research into its mechanistic contributions and therapeutic modulation may revolutionize current strategies for GC prevention, diagnosis, and care.

## Figures and Tables

**Table 1:** Recent advances in microbiota and gastric cancer studies.

Model	Biological System	Study Objective	Key Findings	Ref.
Human clinical observational study	Human gastric mucosal biopsies obtained via endoscopic procedures from 47 patients at various stages of gastric disease: SG (superficial gastritis), AG (atrophic gastritis), GIN (gastric intraepithelial neoplasia), GC (gastric cancer). Human gastric microbiota analyzed using 16S rRNA gene sequencing (targeting regions V3–V4).	To characterize alterations in gastric microbiota associated with different stages of gastric carcinogenesis, identify potential biomarkers, and compare microbiota profiles between cardia and non-cardia gastric cancers.	The study suggested potential microbial biomarkers for early detection. No significant trend in overall microbial richness or diversity across stages. The Shannon index was higher in GIN compared to other groups. The top dominant phyla included *Firmicutes*, *Proteobacteria*, *Bacteroidetes*, *Fusobacteria*, and *Actinobacteria*. Enrichment of oral bacteria (e.g., *Slackia*, *Selenomonas*) increased progressively from gastritis to GC, which may suggest oral flora involvement. Microbiota profiles differed significantly in cardia vs. non-cardia gastric cancer, with higher *Helicobacter* abundance in cardia cancers.	[38]
Human clinical comparative study	Gastric non-malignant and tumor tissue samples from GC patients in China (cardia) and Mexico (non-cardia).	To characterize the human stomach microbiota in gastric cancer patients, compare microbiota between non-malignant and tumor tissues, and assess microbial differences across populations and tissue types.	Microbial diversity and richness were significantly higher in tumor tissues compared to non-tumor tissues. *Helicobacter* was enriched in non-tumor tissues; *Lactobacillus*, *Streptococcus*, *Acinetobacter*, *Prevotella*, and six additional genera were enriched in tumor tissues. Untargeted metabolomics identified 150 discriminative metabolites with higher relative abundance of amino acids, carbohydrates and carbohydrate conjugates, glycerophospholipids, and nucleosides in tumor tissues. Targeted metabolomics revealed that 1-methylnicotinamide and N-acetyl-D-glucosamine-6-phosphate combined serve as robust biomarkers for distinguishing tumor from non-tumor tissue. Correlations suggest that specific bacteria like *Helicobacter* and *Lactobacillus* influence the tumor metabolome, potentially promoting gastric cancer development.	[267]
Human clinical study	Human salivary microbiota characterized through 16S rRNA gene sequencing; a total of 293 patients undergoing endoscopic examination grouped into superficial gastritis (SG), atrophic gastritis (AG), and gastric cancer (GC) stages.	To characterize salivary microbiota changes across progressive stages of gastric carcinogenesis and identify saliva bacterial markers that can be used to detect gastric cancer.	Distinct salivary microbiota profile observed in GC patients with enrichment of proinflammatory taxa, such as *Corynebacterium* and *Streptococcus*. Reduction of bacteria that reduce carcinogenic N-nitroso compounds (e.g., *Haemophilus*, *Neisseria*) in GC. Salivary microbiota profiles distinguished GC from SG and AG with high accuracy (AUC = 0.91). Potential diagnostic biomarkers identified among taxa like unclassified *Streptophyta* and *Streptococcus*. Proposed mechanisms include the accumulation of proinflammatory bacteria and a decline in bacteria that reduce carcinogens, contributing to gastric carcinogenesis.	[150]
Human clinical study (retrospective cohort study)	Gastric mucosal microbiota from patients with gastric cancer (tissues: normal, peritumoral, tumoral).	To investigate the prognostic value of gastric mucosal microbiota in different stomach microhabitats of gastric cancer patients.	Patients with different prognoses showed distinct gastric microbiota compositions and diversity. In peritumoral microhabitats, *Helicobacter* abundance was higher in patients with good prognoses, while *Halomonas* and *Shewanella* were lower. The gastric microbiota association network was more complex in patients with poor prognoses. Predicted microbiota functions varied by microhabitat, notably in peritumoral tissues. Gastric mucosal microbiota alterations may serve as prognostic biomarkers for clinical outcomes in gastric cancer.	[268]
Human clinical study	Human gastric mucosa biopsy samples from 18 gastric cancer (GC) patients and 32 superficial gastritis (SG) patients. Paired tumor and paracancerous tissue samples were collected from GC patients. 16S rRNA gene sequencing for bacterial profiling.	To investigate and compare the gastric mucosal microbiome composition in GC patients and SG patients, assessing differences in bacterial diversity, specific taxa abundance, and predictive functional profiles. To evaluate the microbial dysbiosis index (MDI) as a discriminant metric for gastric-cancer-associated dysbiosis.	GC patients exhibit distinct gastric microbiome profiles compared to SG patients, with significant microbial dysbiosis evident in both tumor and paracancerous tissues. Six bacterial genera were enriched and eighteen were depleted in GC tissues relative to SG. The microbial dysbiosis index (MDI) was significantly higher in GC patients, negatively correlated with microbial diversity (Shannon index), and positively correlated with *Helicobacter* spp. abundance. Functional predictions suggest nitrosating microbial community enrichment in GC patients, implicating microbial metabolism in carcinogenesis. The microbiome differences in GC patients are not limited to tumor sites but also present in adjacent normal tissue, suggesting early microbiome changes in gastric carcinogenesis.	[269]
Human clinical study (case-control study)	Human stomach gastric microbiota in a Korean population involving 556 participants (268 gastric cancer patients and 288 controls). DNA extracted from gastric biopsy samples was analyzed using 16S rRNA gene sequencing to characterize gastric microbiota composition.	The study focused on identifying the relative abundance of specific bacterial species in the gastric mucosa and their association with gastric cancer risk.	Higher relative abundance of *Helicobacter pylori*, *Propionibacterium acnes*, and *Prevotella copri* was significantly associated with increased gastric cancer risk. Higher relative abundance of *Lactococcus lactis* was associated with decreased gastric cancer risk. These bacterial species combined predicted gastric cancer with about 79.7% sensitivity. The study supports microbial profiles as potential diagnostic markers for gastric cancer risk in Koreans.	[270]
Human clinical study	The study involved 227 participants, including 83 gastric cancer patients, 54 gastritis patients, 29 colorectal cancer patients, and 61 healthy controls. Human gut microbiota analyzed via 16S rRNA gene sequencing of fecal samples.	To investigate alterations in gut microbiota composition during gastric cancer progression and evaluate the gut microbiome as a non-invasive predictive marker for gastric cancer diagnosis.	Gut microbiota composition and diversity significantly differ between gastric cancer patients and healthy controls. Random forest model using microbial taxa classified gastric cancer with an AUC of 0.91 and a true positive rate of 0.83 in validation. Gastritis shares some microbiome features with gastric cancer. Chemotherapy reduces microbial abundance and diversity in gastric cancer patients. The genera *Lactobacillus* and *Megasphaera* are significantly associated with gastric cancer and serve as predictive markers.	[271]
Retrospective clinical cohort study	Human gastric cancer patients were classified by body mass index (BMI), specifically low BMI (LBMI) and non-low BMI (NLBMI) groups. Tumor tissues and adjacent normal tissues from these patients were analyzed for intratumoral microbiota, immune cell infiltration, gene expression, and metabolite profiling.	To investigate how BMI relates to intratumoral microbiota and the tumor microenvironment in gastric cancer patients and to identify specific microbial features associated with prognosis in low-BMI patients.	GC patients with low BMI had poorer clinical outcomes and pathological features, and low BMI was an independent risk factor for poor prognosis. 16S rRNA microbial diversity was similar between BMI groups, but 32 bacterial taxa differed, with the genus *Abiotrophia* significantly enriched in LBMI tumors. *Abiotrophia* was negatively correlated with eosinophils and certain genes and positively correlated with others in LBMI tumors. LBMI was linked with increased purine metabolites (guanine, IDP). Low BMI may suppress immune responses and affect chemotherapy efficacy, possibly via changes in intratumoral microbiota and metabolism.	[272]
Human clinical study	Human gastric tissue biopsies from patients with gastritis, intestinal metaplasia, and gastric cancer.	To profile and compare the gastric-epithelium-associated bacterial species among patients with gastritis, intestinal metaplasia, and gastric cancer, aiming to identify additional potential pathogenic bacteria beyond *Helicobacter pylori*.	*Helicobacter pylori* abundance significantly decreased in gastric cancer patients compared to non-cancer patients. *Clostridium* (notably *Clostridium colicanis*), *Fusobacterium* (notably *Fusobacterium nucleatum*), and *Lactobacillus* species were enriched in gastric cancer tissues. *Clostridium* and *Fusobacterium* may serve as diagnostic markers for gastric cancer. The gastric microbiota’s composition differs significantly between cancer and non-cancer groups, suggesting a cancer-specific bacterial signature.	[273]
	Human subjects: 30 gastric cancer patients and 30 healthy controls. Intestinal microbiota analyzed through fecal samples using 16S rRNA gene sequencing technology.	To explore and analyze the composition and characteristics of intestinal microbiota differences between gastric cancer patients and healthy people and to identify specific bacteria associated with gastric cancer.	No significant difference in overall diversity and abundance of intestinal flora between groups. Significant decrease in *Faecalibacterium*, *Bifidobacterium*, and *Subdoligranulum* in gastric cancer patients. Significant increase in *Enterococcus*, *Streptococcus*, and *Bacteroides* in gastric cancer patients. Identified six key intestinal bacterial genera closely related to gastric cancer. Functional pathways enriched in gastric cancer patients’ gut flora include two-component systems, glycolysis/gluconeogenesis, transporters, and others.	[274]

## Data Availability

No new data were created in the present manuscript.

## Abbreviations

The following abbreviations are used in this manuscript:

GC	Gastric cancer
WHO	World Health Organization
IARC	International Agency for Research on Cancer
CNS	Central nervous system
FMT	Fecal microbiota transplantation
MDIs	Microbial dysbiosis indices
CAG	Chronic atrophic gastritis
ROS	Reactive oxygen species
RNS	Reactive nitrogen species
PTEN	Phosphatase and tensin homologue
VEGF	Vascular endothelial growth factor
MNU	N-Methyl-N-nitrosoureas
SCFAs	Short-chain fatty acids
HDAC	Histone deacetylase
TME	Tumor microenvironment
NK	Natural killer cells
DCA	Deoxycholic acid
COX	Cyclooxygenase
DFMO	α-difluoromethylornithine
PUFAs	ω-6 polyunsaturated fatty acids
PGE2	Prostaglandin E2
LPS	Lipopolysaccharide
PAMPs	Pathogen-associated molecular patterns
TLRs	Toll-like receptors
GPCRs	G-protein-coupled receptors
GC/MS	Gas chromatography/mass spectrometry
LC-MS	Liquid chromatography/mass spectrometry
NMR	Nuclear magnetic resonance spectroscopy
TCA	Tricarboxylic acid
GI	Gastrointestinal
PPIs	Proton pump inhibitors
OSCC	Oral squamous cell carcinoma
IBD	Inflammatory bowel disease
PRRs	Pathogen recognition receptors
TAMs	Tumor-associated macrophages
MDSCs	Myeloid-derived suppressor cells
Tregs	Regulatory T cells
pDCs	Plasmacytoid dendritic cells
ICIs	Immune checkpoint inhibitors
IECs	Intestinal epithelial cells
MHC	Major histocompatibility complex class II molecules
TRM	Tissue-resident memory T cells
NAFLD	Nonalcoholic fatty liver disease
ESMO	European Society of Medical Oncology
CSCO	Chinese Society of Clinical Oncology
MSI-H	Microsatellite instability-high
EMA	European Medicines Agency
FDA	Food and Drug Administration
